# Integrating short-read and long-read single-cell transcriptomics of pig pituitary reveals mechanisms of high-altitude hypoxia adaptation

**DOI:** 10.1371/journal.pgen.1012267

**Published:** 2026-08-10

**Authors:** Hai Li, Xudong Wu, Hailong Huo, Xia Zhang, Yiming Guo, Wan Lin, Feidi Wen, Zhijun Zhao, Guiying Zhao, Jinlong Huo

**Affiliations:** 1 College of Animal Science and Technology, Yunnan Agricultural University, Kunming, Yunnan, China; 2 Anhui Provincial Key Laboratory of Livestock and Poultry Product Safety Engineering, Institute of Animal Husbandry and Veterinary Medicine, Anhui Academy of Agricultural Sciences, Hefei, Anhui, China; 3 Yunnan Open University, Kunming, Yunnan, China; 4 Department of Biological and Food Engineering, Lyuliang University, Lvliang, Shanxi, China; 5 Yunnan Animal Husbandry Station, Kunming, Yunnan, China; Xi'an Jiaotong University, CHINA

## Abstract

Acting as the central endocrine hub, the pituitary gland is closely related to the mechanism of adaptation to high-altitude hypoxia. Here, by integratively combining long-read (Oxford Nanopore) and short-read (Illumina) single-cell sequencing approaches, we profiled pituitaries from Diqing Tibetan pigs, inhabiting high-altitude environments (3,200 m) and Diannan small-ear pigs from low-altitude regions (500 m), thereby generating a full-length single-cell atlas, which in turn enabled the identification of molecular mechanisms potentially underlying adaptation to high-altitude hypoxia stress. Systematically delineating pituitary structure and transcriptional dynamics, we profiled 27,339 single cells encompassing 28,932 expressed genes. Leveraging unsupervised clustering coupled with marker-based annotation, we identified ten major cell types. Expression profiling of 20 canonical marker genes revealed pronounced cell-type-specific expression patterns, ten of which were independently validated by immunofluorescence, thus substantiating the accuracy of cell-type annotation. Gene-ontology enrichment analysis further suggested that upregulated genes were predominantly involved in oxidative metabolism and energy production, whereas downregulated genes were significantly associated with protein biosynthesis and translation processes, indicating a functional reprogramming of metabolic pathways. Moreover, comparative analyses between breeds and cell–cell communication analyses highlighted pathway shifts, including broad upregulation of the collagen family and four ligand–receptor pairs that may mediate pituitary intercellular coordination. In parallel, transcription-factor activity analysis nominated several regulators, including EBF3, DBX2, and TCF21, which may collectively contribute to altitude-associated adaptation. Finally, integrating long-read sequencing data revealed widespread transcript isoform diversity, with ~28% novel annotations, indicating considerable isoform complexity in pigs. Our findings delineate cellular heterogeneity, inferred intercellular communication networks, and transcript-isoform diversity in the porcine pituitary, thereby providing a cell-resolved resource for understanding pituitary features associated with high-altitude environments.

## Author summary

The pituitary gland, acting as the central endocrine hub that coordinates growth, metabolism and reproductive function, is plausibly involved in organismal responses to low-oxygen environments. Despite extensive work on systemic and organ-level adaptations to high altitude, detailed knowledge of pituitary cellular composition and transcriptional regulation under chronic hypoxia remains limited. Most prior investigations have relied on bulk tissue assays or short-read sequencing, approaches that mask cell-type heterogeneity and fail to resolve full-length transcript isoforms. Recent advances in single-cell technologies, together with long-read sequencing capable of capturing complete transcript structures, provide an opportunity to link cell-resolved transcriptional states with isoform-level diversity. Using naturally adapted and lowland pig populations as comparative models offers a physiologically relevant framework, since breed-specific differences may reflect long-term evolutionary or developmental responses to altitude. To fill these gaps, we therefore sought an integrative single-cell approach that could characterize pituitary cellular architecture while enabling interrogation of transcript isoform complexity across ecologically contrasting pig populations.

## Introduction

Originating from Xishuangbanna (~500 m), the Diannan small-ear pig (DSE) is adapted to hot and humid lowland environments and is characterized by early maturity, relatively rapid fattening, a small body size, and fine bone structure [1]. In contrast, the Diqing Tibetan pig (DT), mainly distributed in alpine meadows and forest-edge habitats around Shangri-La (~3,200 m), has been exposed to chronic high-altitude environmental pressures, including hypobaric hypoxia, low temperature, stronger ultraviolet radiation, and seasonal nutritional fluctuation. Phenotypically, DT pigs generally exhibit slower growth, stronger environmental tolerance, and distinctive meat-quality traits [2]. The marked altitudinal contrast between DT and DSE pigs provides a valuable natural comparison for exploring pituitary features related to high-altitude adaptation. Together, the distinct ecological backgrounds and breed-specific characteristics of DT and DSE pigs provide a valuable natural framework for investigating altitude-associated pituitary variation in indigenous pig breeds.

The porcine pituitary, which serves as the central regulatory nexus of the endocrine system, secretes a repertoire of pivotal hormones, including growth hormone and gonadotropins, orchestrating physiological homeostasis, metabolic regulation, and reproductive function [3]. Under high-altitude, hypoxic conditions, the pituitary employs a coordinated array of physiological adaptive responses, counteracting the constraints imposed by reduced oxygen availability [4]. Responding to sustained hypoxic stress, pituitary cells undergo pronounced morphological and functional remodeling, recalibrating endocrine output to maintain systemic homeostasis [5]. Notably, hypoxia may potentiate the secretion of specific hormones to promote erythropoiesis and thereby mitigate hypoxia-induced oxygen deficit [6]. Concurrently, hypoxia-associated transcriptional reprogramming within pituitary cells enhances metabolic activity and stress resilience, further reinforcing adaptive capacity [7]. Collectively, these integrated physiological and molecular mechanisms enable the pituitary to sustain normal endocrine and metabolic functions under low-oxygen stress [4]. Elucidating these cellular and molecular dynamics is therefore of critical scientific importance for advancing our understanding of porcine pituitary adaptation to high-altitude hypoxia. However, how the porcine pituitary adapts to long-term altitude-associated environmental pressure at single-cell resolution remains poorly understood. In particular, it is unclear whether high-altitude-associated pituitary remodeling involves changes in cell-type composition, cell-type-specific gene expression, intercellular communication, transcription-factor regulon activity, or transcript isoform usage. Investigating these aspects in high-altitude DT pigs can provide insight into coordinated cell-type-specific transcriptional and isoform-level changes linked to endocrine and microenvironmental adaptation under chronic altitude-related stress.

Conventional bulk RNA sequencing, predicated on total RNA isolated from heterogeneous tissues or cell populations, inherently averages transcriptomic signals, thereby obscuring cellular heterogeneity as well as rare or transient cell states [8]. In contrast, single-cell RNA sequencing (scRNA-seq) enables unbiased [9], high-resolution profiling of individual-cell transcriptomes [10], thereby allowing identification of discrete cell types, dynamic cellular states, stage-specific markers, and their associated regulatory pathways [11–14]. These characteristics are particularly informative for elucidating pituitary responses under altitude-associated environmental stress. Moreover, long-read single-cell sequencing, generating reads approaching full-length transcripts, facilitates accurate reconstruction of splice isoforms, alternative exon usage, and transcript architecture, consequently improving isoform-level quantification and facilitating the detection of allele-specific expression and intracellular structural variants [15–17]. Complementing these capabilities, short-read sequencing, characterized by high accuracy and sequencing depth, reinforces gene-level quantification. Accordingly, short-read data were integrated with long-read data, thereby capitalizing on their complementarity to achieve both precise expression estimates and reliable transcript-structure resolution at single-cell granularity [18,19]. Complementing these capabilities, short-read sequencing, characterized by high accuracy and sequencing depth, reinforces gene-level quantification. Accordingly, short-read data were integrated with long-read data, thereby capitalizing on their complementarity to achieve both precise expression estimates and reliable transcript-structure resolution at single-cell granularity. As a central endocrine organ coordinating metabolism, growth, stress responses, and reproductive function, the pituitary may be sensitive to long-term altitude-associated stress, including hypobaric hypoxia, because systemic adaptation requires coordinated endocrine regulation. We therefore reasoned that altitude-associated environmental pressure may be reflected in pituitary cell composition, cell-type-specific transcriptional programs, intercellular signaling, and transcript isoform structure. Short-read single-cell sequencing resolved cell-type composition, cell-type-specific gene expression, inferred cell-cycle states, and intercellular signaling patterns, whereas long-read single-cell sequencing reconstructed full-length isoforms and characterized transcript structural diversity. By combining these two approaches, we investigated whether altitude-associated pituitary differences occur at both gene-expression and isoform-structure levels. Here, by integrating Illumina short-read and Oxford Nanopore long-read single-cell sequencing, we characterized pituitary cell-type heterogeneity, inferred altitude-associated transcriptional and intercellular signaling changes, and resolved full-length transcript isoform diversity in DT and DSE pigs. This design enabled examination of both gene-level and isoform-level features potentially associated with pituitary responses to ecologically distinct altitudinal environments.

## Results

### Landscape of short-read single-cell transcriptome in porcine pituitary tissues

**Fig 1:**
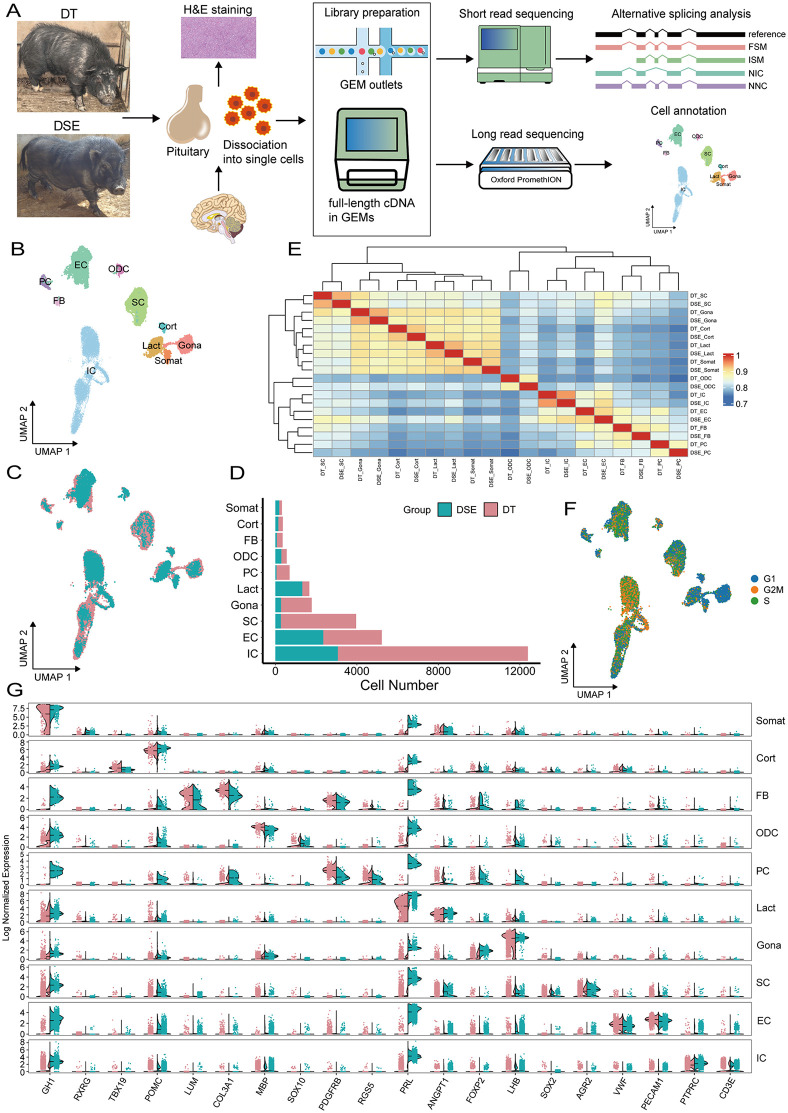
Single-cell transcriptomic profiling delineates the cellular composition and cell cycle dynamics across DT- and DSE-derived pituitary tissues. **(A)** Schematic overview of the scRNA-seq analytical pipeline, outlining key experimental and computational steps. **(B)** UMAP projection resolving annotation of ten transcriptionally distinct cell types, with each point representing an individual cell and clusters annotated through canonical marker genes. **(C)** Combined UMAP embedding of cells derived from DT and DSE pituitaries. Points are colored by tissue origin to illustrate the global distribution and the degree of inter-cohort mixing or segregation. **(D)** Horizontal stacked bar plot summarizing cell counts across the ten annotated cell types. Bar segments represent the relative contribution of DT and DSE to the total cell count for each cell type. **(E)** Heatmap depicting pairwise similarity among biological specimens based on cell-type composition, wherein color intensity reflects correlation coefficient or related similarity metric. Rows and columns are ordered by hierarchical clustering to emphasise inter-sample relationships. **(F)** UMAP embedding with cells coloured according to inferred cell-cycle phase (G1, S, G2/M), which is assigned using established marker-gene scoring, permitting visualization of transcriptomically inferred cell-cycle phase distributions across clusters and tissue origins. **(G)** Pod plot illustrating the expression patterns of selected canonical marker genes across annotated cell types.

Using the 10 × Genomics single-cell workflow, we systematically profiled cellular heterogeneity in pituitary tissues from DT and DSE breeds (Fig 1A). Following enzymatic dissociation, tissues were processed to generate high-quality single-cell suspensions, with erythrocytes efficiently removed to ensure sample purity. The resulting suspensions exhibited an average viable cell concentration of 943 cells/μL, a mean viability of 88.0%, an average cell diameter of 10.7 μm, and a mean doublet rate of 6.1%. After stringent quality control and joint processing, an expression matrix comprising 27,339 cells was retained (DT = 19,292; DSE = 8,047), within which 28,932 genes were robustly detected, thereby establishing a solid foundation for downstream comparative and cell-type–resolved analyses.

Dimensionality reduction and unsupervised clustering were performed using Seurat (v4.3.0), through which ten discrete cell clusters were identified in UMAP space (Figs 1B, S1A and S1B), with their distributions visualized on the same projection (Fig 1C). Given that comprehensive cell-type specific markers for the porcine pituitary remain incompletely defined, cluster annotation was performed by mapping porcine protein-coding genes to their human homologs and by integrating curated marker information from related mammalian pituitary single-cell studies [20–29] and the Human Protein Atlas, thereby enhancing annotation confidence. Based on this integrative strategy and widely accepted marker genes, ten major cell types were annotated (S1C Fig), including corticotrophs (Cort), somatotrophs (Somat), oligodendrocytes (ODC), fibroblasts (FB), pericytes (PC), gonadotrophs (Gona), lactotrophs (Lact), stem cells (SC), endothelial cells (EC) and immune cells (IC). The complete list of marker genes is provided in S1 Table. Absolute cell counts and relative proportions of each cell type across the two porcine pituitaries are summarized in Fig 1D. Of note, immune cells [30] were markedly enriched in the DT relative to DSE, whereas lactotrophs comprised a larger cellular fraction in DSE (S2 Table), a divergence that may reflect an increased propensity for hypoxia-associated inflammatory or immune responses at high altitude, in contrast to low-altitude environments that is more permissive for prolactin-producing cells.

To evaluate similarity across shared cell types, inter-specimen cell-type correlations were computed (Fig 1E). Our results indicated that hormone-secreting cell populations in DT and DSE exhibited high mutual correlation; correspondingly, non–hormone-secreting cell lineages also showed strong mutual correlation, indicating accurate cell-type annotation and a high degree of concordance in cell-type identities between the two pituitaries. To further exclude the potential influence of cell cycle genes associated transcriptional programs on clustering, cell cycle scoring was performed and visualized on the UMAP (Fig 1F). Overall, cells assigned to the G1, S, and G2/M phases displayed a largely uniform spatial distribution, suggesting that cell cycle variation did not substantially dominate the primary clustering structure.

Comparative analysis of UMAP projections combined with G1/S/G2M annotations revealed cell-type-specific differences in cell cycle composition between the two breeds. In DT, several non-hormone-secreting lineages exhibited relatively increased proportions of S- and G2/M-phase cells, suggesting a higher transcriptomically inferred S/G2M-phase signature, whereas certain hormone-secreting populations predominantly occupied G1, indicating a G1-biased cell-cycle signature (S1D Fig). By contrast, in DSE, hormone-secreting cells exhibited higher proliferative proportions of S- and G2/M-phase cells, consistent with a higher inferred S/G2M-phase fraction (S1E Fig). To visualize marker gene expression across clusters, a pod plot was generated (Fig 1G).

### Verify ten cell types using immunofluorescence

**Fig 2:**
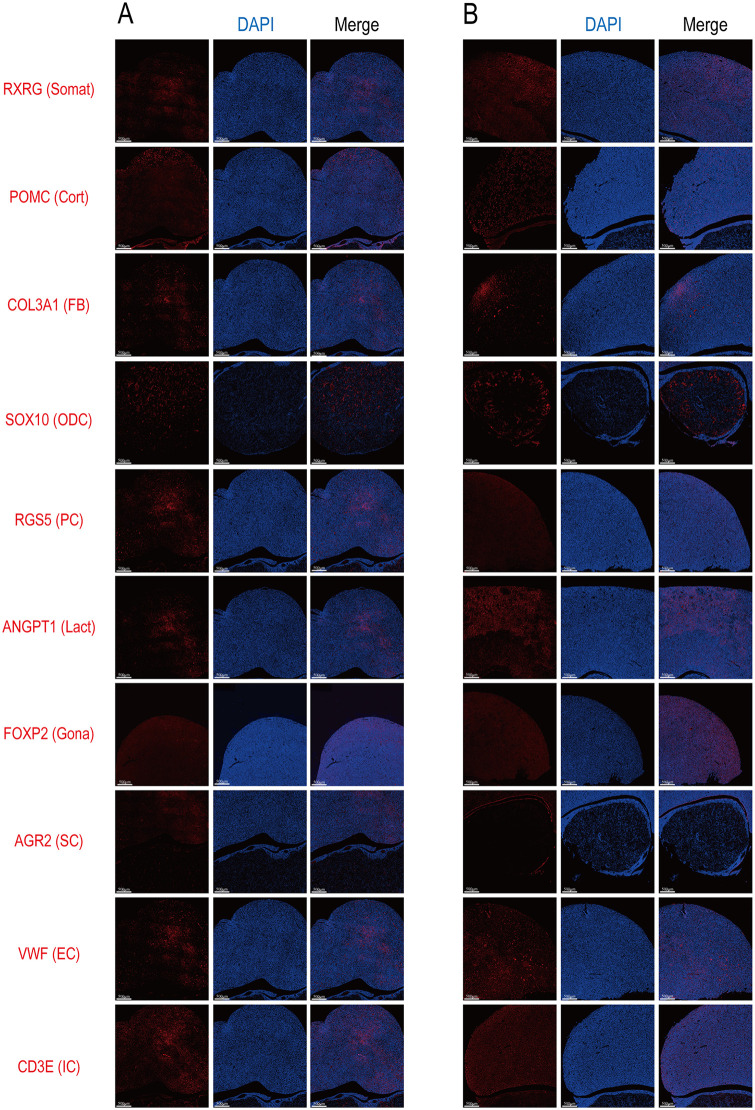
Immunofluorescence validation of pituitary cell identities and spatial distribution. Markers and annotated cell types are listed as follows: POMC (Cort); RXRG (Somat); FOXP2 (Gona); ANGPT1 (Lact); SOX10 (ODC); AGR2 (SC); VWF (EC); CD3E (IC); COL3A1 (FB); and RGS5 (PC). **(A)** Representative confocal micrograph of pituitary tissue from the DT pig, showing the expression and anatomical localization of cell type-specific markers, with nuclei counterstained using DAPI. Images are acquired under identical instrument settings across specimens. Scale bar, 500 µm. **(B)** Representative confocal micrograph from the DSE pig, displaying the expression patterns and spatial arrangement of the same cell type-specific markers as shown in A, likewise counterstained with DAPI and imaged using identical acquisition parameters. Scale bar, 500 µm.

Antibodies targeting cell-type-specific proteins were used for immunofluorescence validation, experimentally confirming the transcriptome-based annotations within the pituitary (S3 Table). The markers and their associated cell types were: POMC (Cort), RXRG (Somat), FOXP2 (Gona), ANGPT1 (Lact), SOX10 (ODC), AGR2 (SC), VWF (EC), CD3E (IC), COL3A1 (FB) and RGS5 (PC). Within the pars distalis, cells expressing POMC, RXRG, FOXP2, and ANGPT1 were organized into endocrine cords embedded within the parenchyma; notably, POMC-positive corticotrophs, which were relatively concentrated in central regions, whereas ANGPT1-positive lactotrophs, which were preferentially enriched in ventral and caudal areas, and FOXP2-positive gonadotrophs were frequently located adjacent to the pars tuberalis. RGS5-positive stem cells, enriched along the marginal zone of Rathke’s cleft, were additionally interspersed throughout the parenchyma. By contrast, SOX10-positive cells localized predominantly to the neurohypophysis and infundibulum, consistent with their glial lineage identity. VWF-expressing endothelial cells formed a permeable sinusoidal capillary network within the pars distalis and were closely apposed to RGS5-positive pericytes; concomitantly, CD3E-positive immune cells were preferentially localized to perivascular and stromal sites, whereas COL3A1-expressing fibroblasts occupied connective septa and perivascular stroma (Fig 2).

### Conservation and heterogeneity of pituitary cells

**Fig 3:**
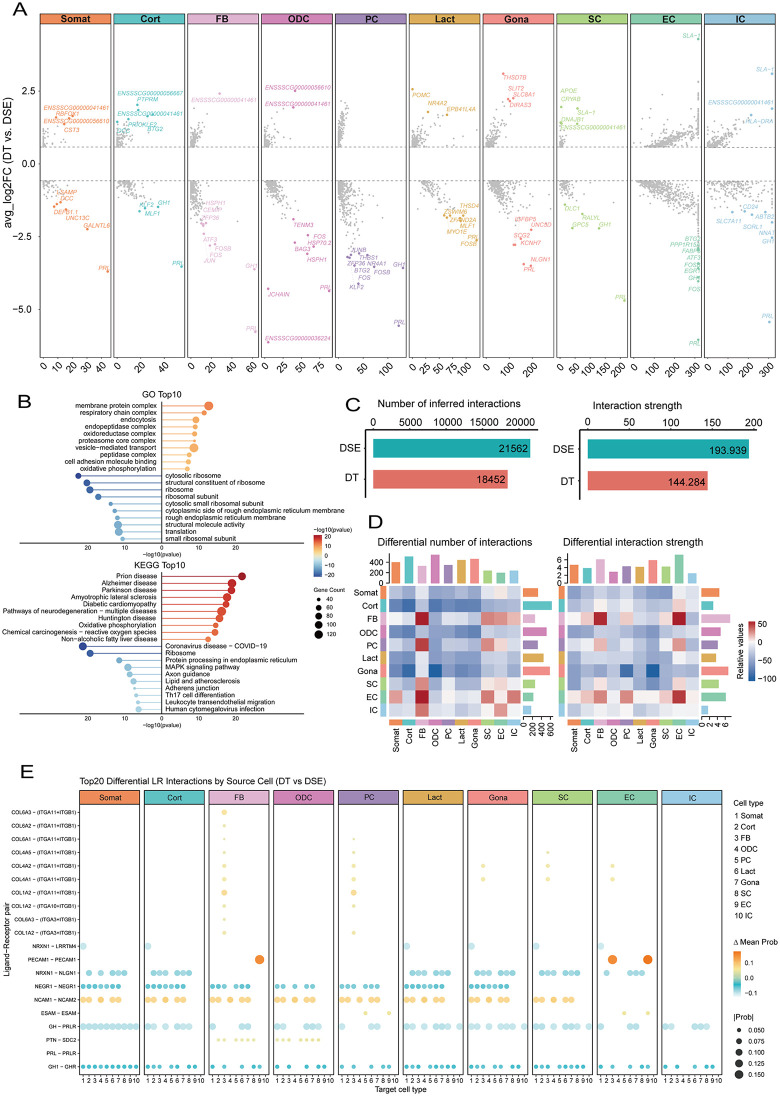
Differences in pituitary cells between DT and DSE. **(A)** Volcano plot showing the top ten DEGs (DT vs DSE). Each point represents a single gene; the y -axis indicates avg_log_2_ fold change. The top 10 genes (ranked by adjusted p-value or effect size) are labeled. **(B)** GO and KEGG pathway enrichment analyses of DEGs. Top ten significantly enrichment terms are displayed as a Lollipop plots, wherein dot size corresponds to gene count and dot color denotes enrichment significance (adjusted p-value) following multiple-testing correction. **(C)** Comparison analysis of total inferred cell-cell interaction counts and aggregate communication strength between DT and DSE. Interaction counts represent the number of interactions across all cell types; aggregate communication strength represents the summed interaction score across all predicted pairs. **(D)** Cell type-resolved differences in interaction counts and signal strength between DT and DSE. For each annotated cell type, heatmap rows represent the differences in the number of inferred interactions and the aggregate signaling strength, allowing the identification of cell types with differential signaling activity. **(E)** Top twenty differentially enriched ligand-receptor pairs between DT and DSE, ranked by differential communication probability, the darker color of the dot, the greater difference.

To systematically delineate transcriptional differences of the pituitaries between DT and DSE, we identified 9,630 differentially expressed genes (DEGs) across pituitary cell populations (S4 Table). Volcano plots indicated the ten most significant DEGs within each cell type between the two groups (Fig 3A). Compared to DSE, 3,628 genes were upregulated whereas 6,002 genes were downregulated in the DT. Collectively, these DEGs were enriched in 8,309 Gene Ontology (GO) pathways (4,108 upregulated and 4,201 downregulated) and 679 Kyoto Encyclopedia of Genes and Genomes (KEGG) pathways (341 upregulated, 338 downregulated) (S5 Table).

GO enrichment analysis suggested that genes upregulated in DT were preferentially associated with mitochondrial respiratory chain complexes, oxidative phosphorylation, membrane protein complexes, oxidoreductase complexes, proteasome core complexes, and endopeptidase complexes, as well as processes like endocytosis, vesicle-mediated transport, and cell adhesion molecule binding (Fig 3B). Conversely, downregulated genes were predominantly enriched in ribosomal and translation-related entries, including cytoplasmic ribosomes, ribosomal subunits, structural molecule activity, and rough endoplasmic reticulum membranes (Fig 3B). KEGG pathways analysis suggested that pathways enriched among DT-upregulated genes were largely dominated by oxidative phosphorylation, neurodegenerative disease-related pathways such as Parkinson’s disease/Alzheimer’s disease, and pathways related to reactive oxygen species. Conversely, pathways enriched among DT-downregulated genes included ribosomes, protein processing in the endoplasmic reticulum, cell adhesion junctions, leukocyte transendothelial migration, Th17 differentiation, MAPK signaling, and various viral infection pathways (Fig 3B). These enrichment patterns suggest that DT pituitary cells may preferentially activate pathways related to mitochondrial ATP production, redox regulation, vesicle-mediated transport, and proteasome-mediated protein quality control. Under altitude-associated stress, these processes could help sustain endocrine cell function by maintaining energy supply, limiting oxidative damage, adjusting membrane or receptor trafficking, and selectively removing damaged proteins. Conversely, the relative downregulation of ribosome- and translation-related pathways may reflect a possible energy-conserving strategy, whereby global biosynthetic activity is reduced while essential maintenance and stress-response pathways are preserved.

Cell–cell communication network analysis indicated that, at the tissue level, DSE exhibited greater communication quantity and stronger communication intensity (Fig 3C), suggesting a more active or tightly coupled intercellular network in DSE. When communication was examined by cell type, however, non-hormone-secreting populations in DT, including endothelial, stromal and immune compartments, showed higher communication quantity and intensity compared with their DSE counterparts (Fig 3D). Detailed per-group metrics, including communication counts (S2A Fig), interaction strength (S2B Fig), overall information flow(S2C Fig), and the distributions of incoming (S2D Fig) and outgoing (S2E Fig) signals for each cell type, are provided in the Supplementary Figure and illustrate that DSE has higher aggregate connectivity while DT concentrates enhanced signaling within non-secretory niches. The higher total communication quantity and strength in DSE reflect the aggregate signaling output across all annotated cell types, which may be influenced by the larger contribution of hormone-secreting populations and broadly distributed endocrine-related interactions. In contrast, the increased communication quantity and strength in DT non-hormone-secreting cells indicate a more localized enhancement of stromal, vascular, endothelial, immune, and pericyte-associated signaling. Thus, DSE may retain a globally more active endocrine communication network, whereas DT may concentrate signaling activity within non-secretory microenvironmental niches potentially related to tissue maintenance and vascular remodeling.

To examine differences in intercellular communication between the two breeds and their potential relevance to high-altitude hypoxia adaptation, we performed differential ligand–receptor analysis on DT and DSE pituitaries and identified 20 ligand–receptor axes showing significant directional changes between breeds. Fourteen axes showed increased expression in DT, including multiple collagen–integrin interactions (COL1A2–(ITGA3 + ITGB1), COL6A3–(ITGA3 + ITGB1), COL1A2–(ITGA10 + ITGB1), COL1A2–(ITGA11 + ITGB1), COL4A1–(ITGA11 + ITGB1), COL4A2–(ITGA11 + ITGB1), COL4A5–(ITGA11 + ITGB1), COL6A1–(ITGA11 + ITGB1), COL6A2–(ITGA11 + ITGB1), COL6A3–(ITGA11 + ITGB1)), and several cell–adhesion/vascular and growth-related pairs (PECAM1–PECAM1, NCAM1–NCAM2, ESAM–ESAM, PTN–SDC2), the largest difference is PECAM1–PECAM1, followed by NCAM1–NCAM2. Six axes were reduced in DT compared with DSE, notably classical endocrine ligand–receptor pairs and neuronal adhesion axes (GH1–GHR, PRL–PRLR, GH–PRLR, NEGR1–NEGR1, NRXN1–NLGN1, NRXN1–LRRTM4). The directional pattern, observed across cell types and after controlling for overall expression differences, indicates coordinated upregulation of extracellular-matrix–integrin and endothelial/adhesion signaling in the DT pituitary, concurrent with attenuation of a subset of hormone-related and synaptic-adhesion interactions. (Fig 3E and S6 Table), highlighting its potential role in mediating high-altitude-associated vascular or intercellular adaptations.

### Transcriptional factor regulatory landscape in the pituitary gland

**Fig 4:**
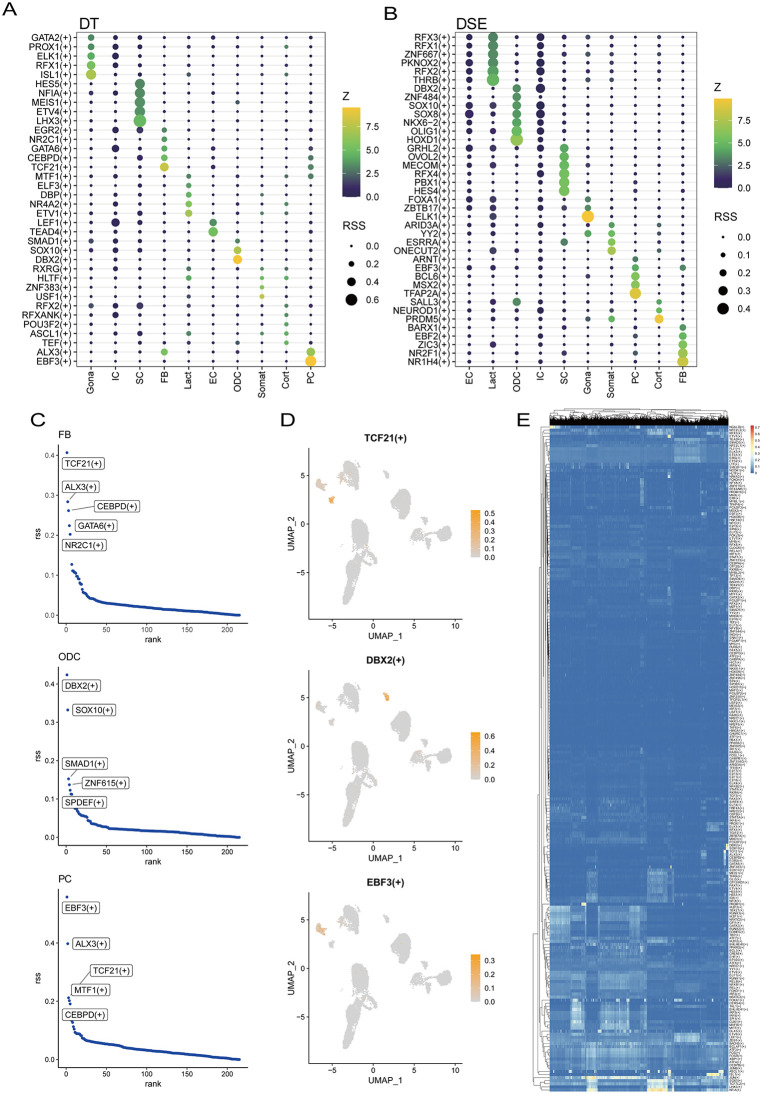
Cell type-resolved regulon activity in pituitary cells. **(A)** Regulon specificity score for DT. Regulon activity is quantified as area-under-the-curve (AUC) values per regulon and per cell using an AUCell-like scoring approach, followed by z-scored normalization; only regulons with |z| ≥ 3 are displayed. Regulons are ranked according to their specificity for DT, with the color scale indicating relative z-scored AUC values. **(B)** RSS for DSE, presented analogously to A, wherein regulons are ordered by specificity to DSE and filtered using the same z-score threshold. **(C)** Cell type-specific regulon ranking in FB、ODC and PC, with regulons ordered by within-cell-type specificity and the top five regulons labeled. **(D)** Spatial localization of TCF21(+), DBX2+) and EBF3(+) cells within the single-cell atlas. TF-positive cells are highlighted to illustrate their cluster distribution and relative abundance across annotated cell types, with color intensity corresponding to RSS. **(E)** AUCell scores for each TF across individual cell.

To systematically evaluate the cell-type-specific transcription factors (TFs) activity underlying pituitary adaptation to high-altitude hypoxia, we calculated and plotted the regulon specificity scores [31] (RSS; z-score normalization, zThreshold = 3) for each annotated cell type in DT and DSE (Fig 4A and 4B, full data in S7 Table). In DT, multiple regulons exhibited particular activity within non-secretory cell populations, including endothelial cells, pericytes, fibroblasts, and immune/stromal-associated cells, such as those expressing EBF3^+^, DBX2^+^, TCF21^+^, TEAD4^+^, LHX3^+^, and RUNX3^+^, thereby implicating these factors in structural remodeling and microenvironmental regulation. In contrast, the DSE pituitary displayed preferential enrichment of distinct regulons within hormone-secreting cells, exemplified by RFX2^+^, ELK1^+^, ONECUT2^+^, and PRDM5^+^, alongside transcription factors such as TFAP2A^+^ and NR1H4^+^ with high specificity in fibroblasts/pericytes, thereby indicating an alternative regulatory strategy focused on homeostasis maintenance and stress responsiveness

To further quantify and visually present the specific regulons’ activity across different cell types in DT, we plotted regulon specificity score plots of regulon activity for the FB, ODC, and PC cell types based on their respective RSS values (Fig 4C; additional cell types in S3A Fig; corresponding DSE results in S3B Fig). These representations delineate cell-type-resolved regulatory signatures, thereby facilitating direct comparisons of transcriptional control across populations. Additionally, on the single-cell atlas we localized the highest-scoring transcription factors for these three cell types (TCF21, DBX2, and EBF3) (Fig 4D), providing a cell-type-resolved visualization of the inferred activity patterns of these candidate regulons. These candidate regulons were prioritized because they showed the highest RSS values in their corresponding cell types, indicating strong cell-type specificity of inferred regulon activity. Given the biological roles of the corresponding cell types, these regulon patterns may provide clues to transcriptional programs associated with pituitary cell identity, tissue remodeling, and microenvironmental regulation under altitude-associated conditions. To experimentally validate the transcriptomic observations for selected candidate regulatory genes, we performed immunofluorescence staining for TCF21, DBX2, and EBF3 in porcine pituitary tissues. Representative immunofluorescence images are shown in S4A–S4C Fig, respectively. Finally, to quantitatively assess TF activity at single-cell resolution, we presented AUCell score heatmaps for each TF (Fig 4E), thereby enabling a fine-grained visualization of relative TF activity across individual cells and reinforcing the cell-specific regulatory heterogeneity observed at the cellular level.

### Characteristics of isoforms identified by long-read single-cell data

**Fig 5:**
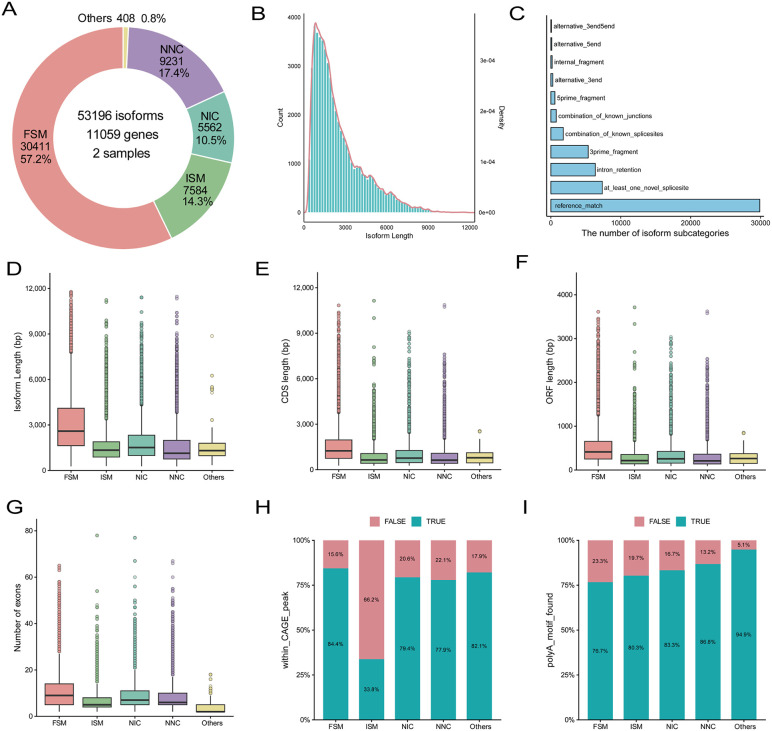
Comprehensive characterization of pituitary isoforms in DT and DSE. **(A)** Pie chart illustrating the percentage of identified isoform types. FSM, Full Splice Match; ISM, Incomplete Splicing Match; NIC, novel in Catalog; NNC, Novel Not in Catalog. **(B)** Histogram displaying the length distributions and corresponding density profiles of all non-redundant isoforms. **(C)** Horizontal histogram representing the structure classifications of detected isoforms. **(D-G)** Quantitative characterization of identified isoforms, including isoform length **(D)**, coding sequence length **(E)**, open reading frame length **(F)**, and exon count **(G)**. **(H)** Overlap of 5’ terminal CAGE caps and 3’terminal poly(A) tails. **(I)** PolyA motifs at transcript 3’ ends.

To comprehensively resolve cellular-level transcriptomic complexity of pituitary tissues in DT and DSE, we employed Oxford Nanopore long-read single-cell sequencing, identifying 53,195 non-redundant isoforms corresponding to 10,987 genes. Leveraging the Ensembl *Sscrofa*11.1 annotation, these isoforms were classified into five categories: full splice match (FSM, 57.2%), representing isoforms that exactly recapitulate annotated splice junctions; incomplete splice match (ISM, 14.3%), exhibiting partial junction concordance; novel in catalog (NIC, 10.5%), corresponding to previously unannotated splice combinations within known genes; novel not in catalog (NNC, 17.4%), arising from unannotated loci; and remaining classes, including fusion or structurally complex genic types (0.8%) (Figs 5A and S5A). Collectively, these data substantially expand the annotated pituitary transcriptome by uncovering a large repertoire of previously unannotated isoforms (S8 Table).

Identified isoforms spanned a broad length distribution ranging from 200 to 12,000 bp, with a pronounced density peak at approximately 900 bp (Fig 5B). Structural analysis revealed that approximately 30,000 transcripts fully matched reference annotation, while more than 7,000 harbored at least one novel splice junction, underscoring extensive alternative splicing diversity (Fig 5C). Comparative assessment across isoform classes further demonstrated that FSM isoforms exhibited longer transcript lengths (Fig 5D), longer coding sequences (CDS; Fig 5E), longer open reading frames (ORFs; Fig 5F), and a greater number of exons (Fig 5G).

Additionally, we systematically evaluated transcription start site overlap with CAGE peaks (within_CAGE_peak; Fig 5H) and the presence of canonical 3′ polyadenylation signals (polyA_motif_found; Fig 5I). The lower 5′-end overlap observed among ISM isoforms may reflect reverse-transcription artefacts, incomplete transcription initiation, or partial mRNA degradation, highlighting technical as well as biological influences on isoform architecture. Taken together, these findings suggest that long-read single-cell sequencing not only enables comprehensive isoform discovery in the pituitary but also facilitates nuanced dissection of transcript structural heterogeneity and its underlying determinants..

### Unique isoform landscapes across pituitary cell classes

**Fig 6:**
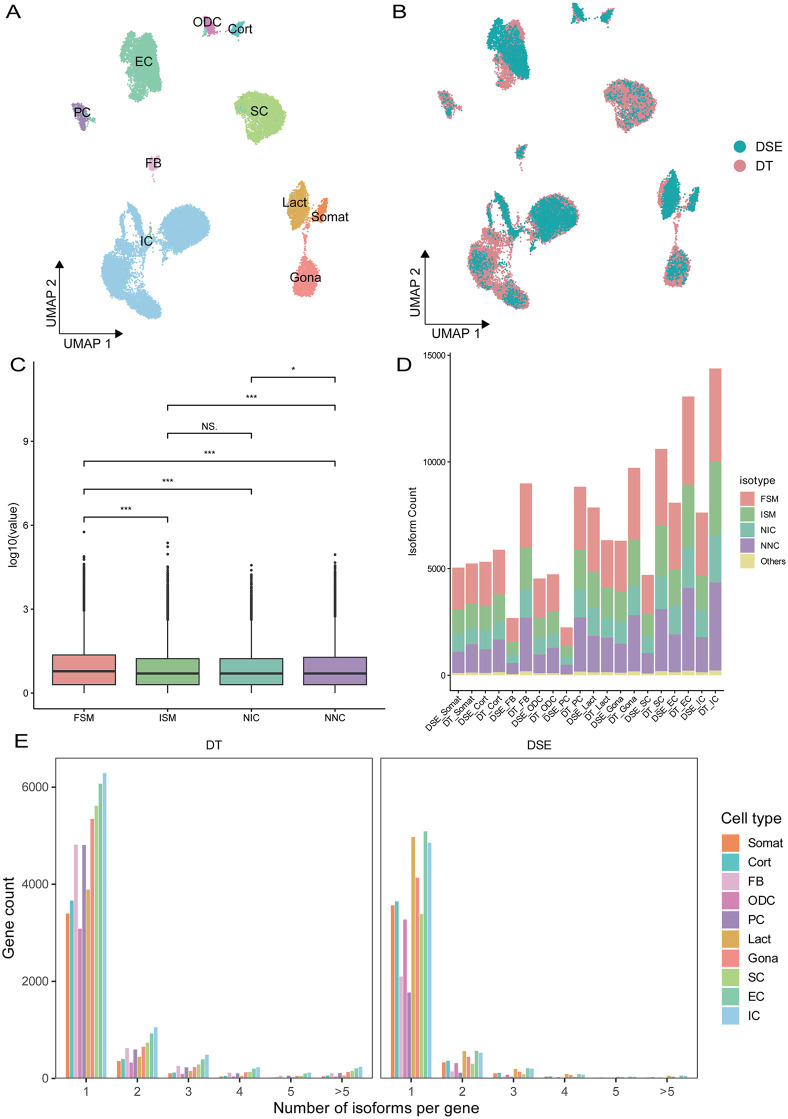
Unique isoform profiles across pituitary cell types in DT and DSE (A) UMAP embedding of the ten transcriptionally defined cell types, with each point representing a single cell colored by cell-type annotation. **(B)** Integrated UMAP of DT and DSE cells, with points colored by origin, illustrating the overall distribution and extent of intergroup mixing or separation. **(C)** Box plots illustrating isoform abundance across different categories, with adjusted *p*-values indicating intergroup differences. **(D)** Bar charts displaying the number of isoform types per cell category. **(E)** Bar charts depicting the number of isoforms per gene in DT and DSE.

To systematically resolve isoform-level transcriptomic diversity in pituitary cells exposed to high-altitude hypoxia, we further characterized isoform expression features across cell types and critically evaluated the capacity of long-read sequencing for detecting low-abundance isoforms. Applying an identical dimensionality reduction and clustering strategy to both short-and long-read data, we analyzed the Nanopore-based single-cell long-read dataset and identified ten major pituitary cell types: corticotropes (Cort), somatotropes (Somat), oligodendrocytes (ODC), fibroblasts (FB), pericytes (PC), gonadotropes (Gona), lactotropes (Lact), stem cells (SC), endothelial cells (EC), and immune cells (IC) (Fig 6A). The spatial distributions of cells from the DT and DSE were coherently projected onto UMAP space (Fig 6B).

Strikingly, newly identified isoforms (NNC and NIC) generally exhibited lower expression levels, thereby plausibly explaining their absence in conventional short-read analyses (Fig 6C). Our findings highlight a unique methodological advantage of third-generation sequencing in detecting low-expression, cell-specific transcripts. Although absolute transcript counts differed substantially among cell types (Fig 6D), the relative distribution of isoform classes (FSM, ISM, NIC, NNC) remained largely conserved between DT and DSE, suggesting that high-altitude hypoxia preferentially remodels cellular function through quantitative modulation of isoform usage and expression abundance rather than through quantitative shift in isoform class composition. Additionally, approximately 80% of expressed genes were dominated by a single isoform (Fig 6E), thereby indicating transcriptomic complexity as being disproportionately concentrated within a limited subset of genes. Notably, genes expressing a single isoform were more prevalent in non-hormone-secreting cells of the high-altitude DT than low-altitude DSE, implying a potential enrichment of transcriptional regulators involved in hypoxia-associated adaptive processes.

### Pituitary cell-type intersections and specificity

**Fig 7:**
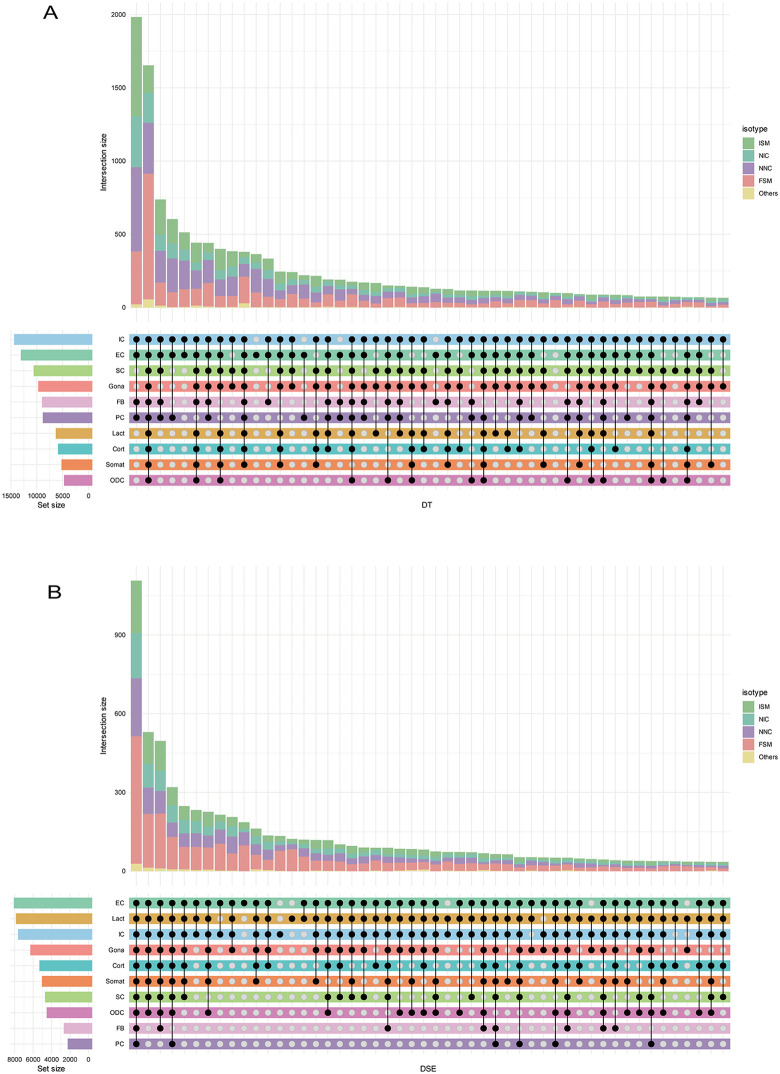
Intersections of transcripts across cell types. **(A)** Top 50 intersections of DT transcripts and isoform-type ratio. **(B)** Top 50 intersections of DSE transcripts and isoform-type ratio.

Comparative Upset plot analyses further revealed a pronounced consolidation of isoform expression in the high-altitude DT, which exhibited substantially larger and more densely concentrated intersections than the low-altitude DSE. Specifically, the three largest intersections in DT encompassed nearly 2,000, 1,600, and 700 isoforms, respectively (Fig 7A), where their counterparts in DSE comprised 1,100, 550, and 500 isoforms, respectively (Fig 7B). Accordingly, the largest and second-largest DT intersections exceeded those of DSE by ~1.8-fold and ~2.9-fold, respectively, while the cumulative total of the top three intersections yielded ~4,300 isoforms in DT compared with ~2,150 in DSE, representing an approximate 2 fold difference, indicated a stronger coordinated and overlapping isoform-level transcriptional response in the high-altitude DT. Nobably, the most prominent DT intersections were predominantly driven by immune cells, endothelial cells, and stem cells, whereas those in DSE were mainly contributed by endothelial cells, lactotropes, and immune cells. Furthermore, DT exhibited a left-skewed Upset distribution characterized by higher peaks and a more gradual decay, consistent with extensive co-expressed or co-regulated of isoforms across multiple cell types under hypoxic stress. In contrast, DSE displayed smaller intersections and a rapidly decaying distribution, reflecting a comparatively dispersed and cell-specific response pattern.

## Discussion

Previous investigations of porcine breeds have predominantly relied on low-resolution methodologies, such as population-level evolutionary analyses and bulk transcriptomic profiling, intrinsically limiting the resolution of cell-type-specific mechanisms [31–33]. Here, we integrated short-read and long-read single-cell transcriptomics to generate a cross-altitude cellular and isoform-level atlas of the porcine pituitary. Although DT and DSE pigs differ markedly in altitude exposure, differences in breed background, growth traits and long-term environmental history may also contribute to the transcriptional and isoform-level variation observed in this study. [18,19]. Leveraging a unified analytical pipeline, we systematically delineated discrete cell populations and their subtype characteristics, while simultaneously reveal subtle differences in isoform utilization, regulatory-network activity, and intercellular communication. Collectively, these findings deepen the molecular and cellular understanding underlying breed-specific hypoxia adaptation, thereby advancing current understanding and providing a rational foundation for breeding strategies to improve hypoxia tolerance and overall health in high-altitude pig populations. Moreover, the cell-type-resolved signatures and candidate targets identified herein could serve as a valuable foundation for subsequent functional validation.

We constructed an integrated single-cell atlas of the pituitary gland by leveraging short-read (Illumina) and long-read (Oxford Nanopore) sequencing technologies across samples derived from distinct altitudes (DT and DSE), systematically characterizing cellular functional heterogeneity and cross-breed differences in cell-type composition. Applying a unified analytical pipeline and quantitatively comparing cell-type abundances, we identified pronounced differences in both cellular identities and their relative abundances across the two breeds. Notably, DT exhibited a marked enrichment of immune cells concomitant with a decreased proportion of lactotropes, a pattern indicative of breed-specific remodeling of pituitary architecture under chronic hypoxic stress. This observation is concordant with transcriptomic results reported by Xie et al [34]., who found hypoxia-associated enrichment of pathways annotated as “neurodegeneration” in pituitary-related expression data, a pattern that the authors interpreted in the context of altered stress and inflammatory signalling. Importantly, enrichment of neurodegeneration-related pathway terms is frequently accompanied by elevated immune/inflammatory signatures in transcriptomic studies, consistent with increased immune-cell representation being a plausible driver of such pathway-level signals. Together, these lines of evidence suggest that the relative increase in immune-cell abundance observed in DT pituitaries may reflect an inflammation-associated remodelling that co-occurs with shifts in endocrine cell composition under chronic hypoxia [35]. Consistently, DT indicated elevated S- and G2/M-phase proportions within immune, endothelial, and stem-cell compartments, collectively reflecting enhanced proliferative activity potentially associated with immune activation or expansion of repair-related cell pools in response to hypoxia [36]. Moreover, cell-cell communication analyses revealed that non-hormone-secreting cells in DT exhibited both higher communication intensity and increased interactions compared with DSE, suggesting more active roles in microenvironmental remodeling and intercellular signaling during adaptation to hypoxia [37,38]. Taken together, these findings support a hypoxia-associated functional reprogramming of the pituitary [39,40], characterized by a shift away from a predominantly hormone-secretion–oriented state toward one prioritizing tissue maintenance, microenvironmental remodeling [41,42], and by increased immune activation and repair-oriented proliferative responses [43–45], ultimately favoring survival and basic homeostasis over energetically demanding endocrine output [46].

Functional enrichment analyses (GO/KEGG) collectively revealed a model of metabolic reallocation. Specifically, the upregulation of respiratory-chain complexes and oxidative phosphorylation pathways, accompanied by the enrichment of oxidoreductase activity and ROS-related processes, suggested that DT pituitary cells may recalibrate mitochondrial capacity and electron-transport efficiency to maintain ATP production under oxygen-limited conditions [47,48], albeit concomitantly elevating ROS generation and thereby necessitating compensatory antioxidant and repair responses [49,50]. In parallel, the coordinated enrichment of proteasome, endopeptidase, and vesicle-mediated transport-related pathways indicated increased selective protein turnover and membrane-protein remodeling [51–53], thereby facilitating the removal of oxidatively damaged proteins and enabling rapid reconfiguration of receptors and ion channels in response to changing microenvironmental cues [54,55]. By contrast, the broad suppression of ribosome- and translation-related pathways reflected a canonical energy-saving strategy [56,57], whereby global protein synthesis was downregulated to reallocate energetic resources toward maintenance of membrane potential, selective secretion, and cellular repair [58]. Consistently, the downregulation of ER protein-processing pathways further suggested a deliberate reduction in nascent protein load, thereby mitigating ER stress [59]. Notably, apparent enrichment of pathways classically associated with neurodegenerative disease likely reflected shared molecular themes, including mitochondrial dysfunction, oxidative stress, and proteostasis imbalance [60,61], whose signatures may represent adaptive or compensatory molecular states. Collectively, the observed enrichment landscape, characterized by restrained global translation and inflammation signaling yet enhanced mitochondrial performance and selective proteostatic capacity, is concordant with established cellular strategies for long-term adaptation to hypoxic stress.

Ligand–receptor differential analysis revealed a coordinated upregulation of extracellular-matrix–integrin axes and endothelial/adhesion signaling in DT, with the collagen family broadly elevated and the PECAM1–PECAM1 interaction showing the largest relative increase [62,63]. Collagen-family–integrin signaling, being central to matrix assembly and mechanoresponsive cues, may support structural remodelling of the pituitary stroma [41,64], whereas heightened PECAM1–PECAM1 and ESAM–ESAM signaling [65,66]-both endothelial adhesion modules-plausibly reflect reinforced endothelial junctional integrity, altered leukocyte trafficking, and increased mechanotransductive capacity in the DT vasculature [63,67]. The NCAM1–NCAM2 axis, by modulating homophilic and heterophilic adhesion and by supporting cell survival signalling, might contribute to strengthened cell–cell cohesion and paracrine stability [68,69] across endocrine and non-endocrine compartments. Meanwhile, elevated PTN–SDC2 signalling, consistent with growth-factor–mediated matrix remodelling and angiogenic modulation, may promote perivascular niche reconfiguration and local trophic support [70,71]. Taken together, and noting the concurrent activation of endothelial- and pericyte-associated regulons (including TEAD4 [72], RUNX3 [73] and TCF21 [74]), these coordinated changes are consistent with a program that favors reinforcement of vessel–matrix interactions and microvascular stability, thereby potentially improving local oxygen delivery and supporting cell survival under chronic hypoxia [62,64]. By contrast, several classical endocrine and synaptic-adhesion axes were relatively attenuated in DT [75]. The observed reduction in GH1–GHR, PRL–PRLR and related growth-hormone/prolactin signalling pairs may indicate a reallocation of energetic and biosynthetic capacity away from constitutive hormone synthesis and secretion [76,77]. Similarly, downregulation of synaptic/adhesion-like interactions (NEGR1–NEGR1, NRXN1–NLGN1, NRXN1–LRRTM4) may reflect a diminished reliance on synapse-like contacts and rapid neurotransmission-type signalling within the pituitary tissue [67,78,79], consistent with an energy-conserving phenotype. Collectively, the reciprocal pattern-enhanced matrix, endothelial and adhesion signalling paired with reduced endocrine and synaptic-adhesion communication, which suggests a hypoxia-associated reprogramming in which tissue maintenance, vascular stability and selective paracrine support are prioritized over energetically costly secretory programs [64].

Regulons that are selectively upregulated and specifically active in non-secretory cells of DT, including TEAD4, RUNX3, TCF21, LHX3, and EBF3, collectively delineated the activation of several key biological programs. TEAD4 [72], RUNX3 [80] and TCF21 [81,82], which have been extensively implicated in vascular and stromal remodeling, likely promote angiogenic and matrix-remodeling processes; LHX3, a transcription factor essential for pituitary development and lineage maintenance [83], could serve as a dual function role under hypoxia by preserving essential pituitary cell identities while initiating tissue reconstruction programs; and EBF3, together with other stress-responsive regulators, may contribute to adaptive cell-fate control and stress-mitigation responses [84]. Our results suggest that regulon programs governing cell-identity regulation, stromal and vascular remodeling, and cellular stress adaptation are preferentially in DT. By contrast, DSE largely retains regulon activity associated with secretory homeostasis and endocrine function. Specifically, RFX2 is linked to cellular differentiation and ciliary functions [85]; ELK1, an immediate-early transcription factor operating downstream of MAPK signaling in stress responses [86]; ONECUT2 regulates angiogenesis-related genes under hypoxic conditions [87]; and PRDM5, a regulator of extracellular matrix gene programs [88]. Overall, our results indicated that DT preferentially enhanced tolerance to chronic hypoxia by activating vascular and matrix remodeling alongside metabolic and protein homeostasis networks in non-secretory cells, whereas DSE maintained a more canonical response mode dominated by endocrine and extracellular matrix homeostasis. a pattern consistent with its relatively higher hormone-cell proliferation and secretory activity. Collectively, these findings support a model in which DT undergoes a functional reallocation of pituitary programs toward vascular remodeling and cell-identity maintenance, albeit at the expense of secretion-oriented pathways, thereby favoring tissue survival under hypoxia.

Long-read sequencing revealed that approximately 28% of the isoforms annotated in the porcine pituitary were previously unannotated NIC or NNC transcripts, indicating that current reference annotations still incompletely capture pituitary transcript diversity. Consistent with previous observations that many novel isoforms are lowly expressed and highly cell-type-specific [89,90], these transcripts may represent a regulatory layer largely missed by gene-level analyses. Compared with DSE, DT showed markedly larger isoform-intersection sizes [18], suggesting broader transcript sharing or more coordinated isoform deployment across pituitary cell types [91]. This pattern may reflect coordinated isoform-level regulatory responses to high-altitude-associated pressures, through which specific cell populations adjust coding potential, untranslated regions, transcript stability, protein-domain composition or subcellular targeting in response to hypoxia-related demands [92]. In particular, the preferential use of a single dominant isoform in DT non-secretory cells may indicate a more streamlined isoform-use pattern that may help maintain transcriptional stability while reducing regulatory complexity [93], supporting essential cellular functions while limiting metabolic cost. Together, these findings suggest that isoform-level regulation provides an additional dimension of altitude-associated pituitary remodeling, enabling cell-type-specific fine-tuning of metabolic, proteostatic and microenvironmental responses without necessarily altering total gene abundance.

Together, this study provides a cellular and isoform-level framework for understanding pituitary variation in indigenous pigs living at different altitudes. However, the DT-DSE comparison represents a natural ecological and breed-level contrast, in which hypobaric hypoxia co-occurs with differences in temperature, ultraviolet exposure, nutrition, husbandry history, growth traits, and genetic background. The molecular features identified here should therefore be interpreted as high-altitude-associated signatures in native breed contexts, rather than as oxygen-specific effects alone. Because the present datasets were generated from a limited number of animals, larger cohorts, reciprocal environmental designs, physiological measurements, and targeted experimental validation will be important for further testing the causal roles and broader relevance of the candidate genes, pathways, ligand-receptor pairs, regulons, and isoforms highlighted by this atlas.

## Materials and methods

### Ethics statement

The animal study was reviewed and approved by the Life Science Ethics Committee of Yunnan Agricultural University (approval number YNAU202403050). All experimental protocols were conducted in accordance with the approved guidelines.

### Animals and samples

For single-cell sequencing, pituitary tissues were collected from one 18-month-old male Diqing Tibetan pig (DT) originating from Shangri-La, Diqing Prefecture, Yunnan, China, at an altitude of approximately 3,200 m, and one 18-month-old male Diannan small-ear pig (DSE) originating from Xishuangbanna, Yunnan, China, at an altitude of approximately 500 m. Additional pituitary tissues from DT and DSE pigs of comparable age and body weight were used for histological and immunofluorescence analyses. Following craniotomy performed by a licensed veterinarian, the pituitary glands were aseptically excised. After meticulous removal of adherent connective tissue and fascia, the pituitary glands were preserved at 4°C in tissue-storage solution for subsequent single-cell isolation. In parallel, pituitary glands obtained from pigs with comparable body weight and raised under identical conditions were collected and fixed in 4% paraformaldehyde for histological analyses.

### Histological analysis

Pituitary specimens were dehydrated through a graded ethanol series, cleared in xylene, and embedded in paraffin. The resulting tissue blocks were sectioned at 4–6 μm using a Leica RM2016 rotary microtome (Leica, Shanghai, China). Sections were subsequently stained with hematoxylin and eosin (H&E; Servicebio, Wuhan, China) to visualize histological architecture and cellular organization. Whole-slide images of the pituitary were acquired with a Pannoramic MIDI FL scanner (3DHISTECH, Budapest, Hungary) for high-resolution morphological analysis.

### Immunofluorescence analysis of pituitary tissues

Paraffin-embedded pituitary sections were deparaffinized in xylene and rehydrated through a graded ethanol series. Antigen retrieval was performed in EDTA buffer using microwave heating. After cooling, sections were washed with PBS and blocked with BSA. Primary antibodies were applied and incubated overnight at 4°C. CY3-conjugated secondary antibodies (1:500 dilution) were incubated in the dark. Sections were subsequently washed with TBST, counterstained with DAPI, mounted, and examined under a fluorescence microscope.

### Single-cell suspension preparation

Pituitary tissues were washed twice with DMEM to remove residual preservative, minced into 2–3 mm³ fragments, and enzymatically digested at 37°C. The resulting cell suspension was filtered through a 40 µm nylon cell strainer and centrifuged at 300 g for 5 min at 4°C. Pelleted cells were resuspended in an ice-cold PBS-BSA. Erythrocyte contamination was addressed by incubating the suspension with Red Blood Cell Lysis Buffer (Miltenyi Biotec) for 10 min at room temperature, with volume adjusted empirically based on visible hemolysis. Cell concentration, viability, and aggregate content were assessed using trypan blue staining and a Countess II FL automated cell counter. Aggregate rates were maintained below 5%, and cell viability exceeded 95%. Residual dead cells were subsequently eliminated using the Dead Cell Removal Kit (Miltenyi Biotec).

### Single-cell transcriptome library construction

Single-cell transcriptome libraries were generated using the Chromium Single Cell 3′ Reagent Kit v3.1 (10 × Genomics) according to the manufacturer’s instructions. Prior to encapsulation, cell suspensions were evaluated by 0.4% Trypan Blue exclusion using a Countess II automated cell counter (Thermo Fisher Scientific) and adjusted to a viability exceeding 90% and a concentration of 800–1,200 cells·µL^−1^. Cells were then co-partitioned with barcoded gel beads and reaction master mix in a Chromium Controller to generate Gel Bead-in-Emulsions (GEMs). Following GEM disruption, nucleic acids were purified using Dynabeads MyOne SILANE (Thermo Fisher Scientific) and amplified to generate cDNA libraries, which were subsequently quantified on an Agilent 2100 Bioanalyzer using the High Sensitivity DNA Kit (Agilent Technologies).

### Illumina single-cell sequencing

Short-read sequencing data from single-cell transcriptome libraries were processed using CellRanger (v9.0.1). Following adapter trimming and the removal of technical artifacts, reads were demultiplexed and aligned to the porcine reference genome (*Sscrofa*11.1, Ensembl release 113), thereby enabling the evaluation of alignment metrics and mapping quality. Low-quality, unmapped and multi-mapped reads, as well as PCR duplicates were subsequently excluded. Gene-level transcript abundances were then generated by collapsing reads according to unique UMIs and cell barcodes, ultimately yielding a UMI-count matrix suitable for downstream expression profiling and functional analyses.

### Nanopore single-cell sequencing

Following rigorous quality control, cDNA amplicons were subjected to end-repair and 3′ adenylation using NEBNext Ultra II reagents (New England Biolabs). Sequencing adapters were subsequently ligated using the SQK-LSK114 Ligation Sequencing Kit (Oxford Nanopore Technologies), and excess adapters were removed via magnetic-bead purification with AMPure XP (Beckman Coulter). The resulting libraries were quantified on a Qubit 4.0 fluorometer (Thermo Fisher Scientific) and loaded onto a PromethION R10.4.1 flow cell (Oxford Nanopore Technologies) for real-time, single-molecule nanopore sequencing.

### Illumina short-read data preprocessing

Raw Illumina short-read data were preprocessed with CellRanger v9.0.1 (10 × Genomics) to remove sequencing adapters and low-quality bases. The resulting adapter-trimmed reads were aligned to the *Sus scrofa* reference genome (*Sscrofa*11.1, Ensembl release 113) using STAR v2.7.10a. Subsequently, for accurate quantification, we excluded low-quality, unmapped, multimapped reads, as well as PCR duplicates. Gene-by-cell expression matrices were then constructed by enumerating UMIs and cell barcodes via the CellRanger’s count pipeline, with transcript abundance quantified against Ensembl-annotated genes.

### Nanopore long-read data preprocessing

Raw Nanopore signal data (FAST5 files) were base-called with Guppy v6.0.7 with the high-accuracy model to maximize read-level fidelity. Cellular barcodes were subsequently recovered through identification of predefined flanking sequences, with barcode assignment further refined by adaptively calibrating the Levenshtein edit-distance threshold (1–3) according to cross-correlation analyses between Illumina-derived cell barcodes and their matched gene-expression profiles. The resulting demultiplexed cellular barcodes and UMIs were then appended to each read header in the output FASTQ files, enabling unequivocal attribution of long reads to individual cells while simultaneously preserving the integrity of the original sequence information.

### Cell clustering and annotation

To delineate pituitary cellular heterogeneity, functional states, and tissue organization, clustering and cell-type annotation were conducted. The raw count matrix was imported and subjected to stringent quality-control filtering, excluding cells with a mitochondrial transcript fraction **≥**10%, retaining only those with at least 200 detected genes, and preserving genes expressed in a minimum of three cells. Potential ambient contamination was subsequently assessed using the decontX function of celda (v1.24.0),with cells exhibiting contamination scores **≥** 0.2 being discarded. Doublets and multiplets were then identified and removed using scDblFinder (v1.22.0) to improve the reliability of downstream analyses. The remaining expression counts, after normalization with NormalizeData, were used to identify highly variable genes, enriching biological signal while attenuating technical noise. Dimensionality reduction was performed with PCA (RunPCA) followed by UMAP (RunUMAP) for low dimensional embedding. A shared nearest-neighbor graph (FindNeighbors) was constructed, and cluster-specific marker genes were determined using FindAllMarkers for downstream annotation.

### Cell cycle analysis

Cell-cycle dynamics were initially assessed by applying the curated phase-specific gene sets provided in Seurat v4.4.0, with cell-cycle scores computed using the CellCycleScoring function. Each cell was subsequently assigned to the G1, S, or G2/M phase based on the relative enrichment of corresponding gene signatures. The resulting cell-cycle states were then projected onto the UMAP embedding and visualized by coloring cells according to their assigned phase, facilitating intuitive interpretation of proliferative heterogeneity across cell populations.

### Functional enrichment analysis

Differentially expressed genes (DEGs) between the two pig breeds were identified using the FindMarkers function in Seurat v4.4.0, applying the Wilcoxon rank-sum test as the underlying statistical framework. Genes meeting an adjusted *P*-value < 0.05 and an absolute log_2_ fold change > 1 were retained as significant. Functional enrichment analysis was subsequently performed using clusterProfiler (v4.16.0), within which GO and KEGG pathways were interrogated for over-representation via hypergeometric testing. Following correction for multiple comparisons, GO terms and KEGG pathways with adjusted *P*-value < 0.05 were deemed signicicantly enriched. The resulting enrichment profiles were then visualized using ggplot2 v4.0.0.

### Cell-cell communication inference

To systematically interrogate global intercellular signaling within the porcine pituitary, we performed cell-cell communication analysis using CellChat (v2.2.0). Porcine genes were first mapped to their human orthologs to leverage shared ligand-receptor annotations, and group-wise communication networks were inferred for each breed. Porcine genes were converted to human orthologs using Ensembl BioMart. Orthologs were retained only when a confident mapping relationship was available, resulting in 14,985 mapped genes among 28,932 porcine genes (51.79%). Unmapped genes were excluded from human-reference-dependent analyses, including marker-based cell-type annotation, CellChat ligand-receptor inference, and pySCENIC regulon analysis. For many-to-one or one-to-many ortholog relationships, the best-supported ortholog was retained only when a clear assignment was available based on orthology confidence and sequence similarity; otherwise, ambiguous mappings were excluded to reduce potential functional misassignment. Differential interaction patterns between DT and DSE were subsequently conducted with CellChat’s netVisual_diffInteraction function, facilitating a global comparison of signaling architectures. In parallel, we quantitatively compared inferred communication probabilities for shared ligand-receptor pairs between breeds and ranked them by the magnitude of their differential signaling to extract the top 20 most markedly altered ligand-receptor interactions. Cell-cell communication analysis was performed using CellChat v2.2.0. Porcine genes were converted to human orthologs before matching to the CellChat human ligand-receptor database. Only ligand-receptor pairs in which both ligand and receptor genes were successfully mapped to human orthologs were retained, whereas unmapped or ambiguously mapped genes were excluded. Overexpressed ligands and receptors were identified using CellChat’s default expression-filtering and statistical framework, and no additional manual expression threshold was applied. Communication probabilities were inferred using permutation-based testing. Differential ligand-receptor pairs between DT and DSE were ranked according to the absolute difference in inferred communication probability, and pairs with adjusted P-value < 0.05 were considered significant.

### Gene regulatory network analysis

Gene regulatory networks (GRNs) were inferred from single-cell expression matrices, mapped between porcine and human orthologs, using pySCENIC (v0.12.1). The analytical workflow comprised three sequential stages. First, during the GRN inference step, co-expression relationships between transcription factors (TFs) and candidate target genes were derived from the expression matrix, yielding a TF-target interaction matrix that seeded downstream module detection. Second, in the context motif enrichment stage, we refined these co-expression modules by TF-binding motif enrichment analysis using RcisTarget databases, thereby assigning TFs to modules only when DNA-binding evidence supported the regulatory links and filtering putative false positives. Third, we quantified regulon activity at the single-cell level using AUCell, which computed an area-under-the-curve (AUC) score reflecting the enrichment of each regulon’s target genes within a cell-specific ranked expression profile. High-activity regulons were identified using default thresholds, producing a regulon-by-cell AUC matrix for downstream visualization and comparative analysis.

### Detection and quantification of isoforms

To ensure the generation of high-fidelity barcodes and the accurate quantification of pituitary cell subtypes from long-read data, we first identified adapter and barcode loci with BLAZE (v2.0.7), thereby yielding precise barcode coordinates. Subsequently, Barcode-UMI sequences extracted from long-read data were systematically cross-validated against cell annotations derived from short-read data, thereby minimizing the risk of misassignment. The resulting validated reads were then aligned to the Sus scrofa reference genome (*Sscrofa*11.1) using minimap2 (v2.24). Genome-mapped reads were subsequently polished and hierarchically clustered into consensus transcript assemblies. Redundant isoforms were collapsed during this process, whereas truncated 5′ isoforms and degradation products were subsequently eliminated using cDNA_Cupcake, ultimately yielding a high-confidence repertoire of full-length isoforms.

### Isoform classification and filtering

To ensure transcript accuracy and structural completeness, identified transcripts were systematically classified and subjected to rigorous quality control with SQANTI3 (v5.2.0), through comparison with the reference genome. SQANTI3-derived isoform classifications were extracted and visualized across cell types using ComplexUpset (v1.3.6). FSM and ISM transcripts were categorized as known isoforms, whereas NIC and NNC were classified as novel isoforms of annotated genes. Fusion, genic, intergenic, and antisense transcripts were collectively grouped as novel-type isoforms. To maximize confidence in downstream analyses, we excluded isoforms harbouring non-canonical splice junctions, predicted targets of nonsense-mediated decay (NMD), or 3′ termini deviating by ≥100 bp from annotated polyadenylation sites. Starting from 110,214 initially classified isoforms, we first removed 14,200 isoforms containing non-canonical splice junctions, leaving 96,014 isoforms. We then removed 9,922 isoforms flagged at the RTS-filtering step, followed by removal of 23,860 isoforms predicted as NMD targets, leaving 62,232 isoforms. Finally, we removed 9,036 isoforms with 3′ terminal deviations of ≥100 bp from annotated polyadenylation sites, resulting in a final high-confidence set of 53,196 isoforms. Altogether, this multi-tiered filtering strategy generated a high-confidence catalogue of full-length isoforms.

## Supporting information

S1 Fig Overview of clustering, cell-cycle assignment, quality control, and top differentially expressed genes. (**A**) Combined UMAP embedding of single cells showing 36 annotated clusters. Each point represents a single cell and is colored by cluster identity to illustrate the global cellular landscape and relative cluster sizes. (**B**) Boxplots summarizing quality-control metrics for each annotated cell type: number of UMIs (nCount_RNA), number of detected genes (nFeature_RNA), and percentage of mitochondrial transcripts (percent.mt). For each metric, boxes indicate median and interquartile range (IQR), whiskers extend to 1.5 × IQR, and individual cells are shown as points. (**C**) Heatmap of the top four differentially expressed genes (DEGs) for each annotated cell type. (**D**) UMAP of cells from the DT sample, colored by inferred cell-cycle phase (G1, S, G2/M). Cell-cycle phase is assigned using canonical S- and G2/M-phase marker genes and the standard scoring procedure (see Methods). The panel highlights the distribution of cycling versus non-cycling cells within DT. (**E**) UMAP of cells from the DSE sample, colored by inferred cell-cycle phase (G1, S, G2/M), generated and annotated as in (B) to allow direct comparison of cell-cycle composition between DT and DSE.
(TIF)

S2 Fig Comparative overview of inferred cell–cell communication in DT and DSE. (**A**) Chord plot summarizing the number and aggregated strength of predicted cell–cell interactions in the DT sample. (**B**) Chord plot summarizing the number and aggregated strength of predicted cell–cell interactions in the DSE sample. (**C**) Overall information flow per signaling pathway across the combined network. (**D**) Incoming signaling (DT and DSE). (**E**) Outgoing signaling (DT and DSE).
(TIF)

S3 Fig Regulon specificity ranking for DT and DSE cell types. (**A**) Ranked regulon-specificity plots for the remaining annotated cell types in the DT group. For each recipient cell type, regulons are ordered along the x-axis by decreasing RSS and the corresponding RSS value is plotted on the y-axis. Higher RSS indicates greater specificity of a given regulon to the focal cell type. The top five regulons (n = 5) with the highest RSS for each cell type are labeled on the plot. (**B**) Ranked regulon-specificity plots for all annotated cell types in the DSE group. Plotting conventions mirror panel (A): regulons are ordered by decreasing RSS on the x-axis, RSS values are shown on the y-axis, and the top five regulons (n = 5) per cell type are labeled.
(TIF)

S4 Fig Immunofluorescence validation of candidate regulatory genes in porcine pituitary tissue. (**A**) Immunofluorescence staining of TCF21. (**B**) Immunofluorescence staining of DBX2. (**C**) Immunofluorescence staining of EBF3. Nuclei were counterstained with DAPI. Scale bars are indicated in the images.
(TIF)

S5 Fig Proportions of transcripts of DT and DSE. (**A**) Pie chart showing the proportional composition of transcript isoform classes in DT. Each sector represents one isoform class (FSM, ISM, NIC, NNC, and “others”), with sector labels giving the percentage of total expressed transcripts and the absolute transcript count (n) in parentheses. The “others” category includes transcripts not assigned to FSM, ISM, NIC, or NNC classes. (**B**) Pie chart showing the proportional composition of transcript isoform classes in DSE. Conventions are identical to panel A: sectors correspond to FSM, ISM, NIC, NNC, and “others,” with percentage and absolute count (n) reported for each sector.
(TIF)

S1 Table Marker genes for annotated cell clusters. (XLSX)

S2 Table Cell-lineage and subtype counts and proportions. (XLSX)

S3 Table Antibody metadata. (XLSX)

S4 Table Differentially expressed genes (DT vs DSE). (XLSX)

S5 Table Functional enrichment of differentially expressed genes. (XLSX)

S6 Table Predicted ligand–receptor interactions for DT and DSE. (XLSX)

S7 Table Regulator specificity scores by cell type. (XLSX)

S8 Table Isoform-level annotations. (XLSX)

S9 Table Isoform counts and isotype distribution across cell types. (XLSX)
