# Supplementary material for: Integrating short-read and long-read single-cell transcriptomics of pig pituitary reveals mechanisms of high-altitude hypoxia adaptation
Source: PLoS Genet. 2026 Aug 10;22(8):e1012267. doi: 10.1371/journal.pgen.1012267 (PMC13472502; doi:10.1371/journal.pgen.1012267)
Supplement: Supplementary file 1 [file pgen.1012267.s016.docx]

Dear PLOS Genetics Team,

On behalf of all authors, we are pleased to resubmit a revision of our manuscript entitled **“Integrating short-read and long-read single-cell transcriptomics of pig pituitary reveals mechanisms of high-altitude hypoxia adaptation”** (PGENETICS-D-26-00113) to ***PLOS Genetics***.

We sincerely appreciate the time and effort you have dedicated to providing valuable and insightful feedback on our manuscript. We also appreciate your interest in our work and your encouragement for us to revise the manuscript.

Please find more details in our point-to-point responses. We hope that this revision has adequately addressed all the Reviewers’ comments and that the manuscript now meets the standards of ***PLOS Genetics*** for publication. Thank you very much for your consideration.

**Content**

**Editor’s Comment and Author’s Response:	3**

**[Reviewer #1’s Comments and Author’s Responses](#_Toc221025269)**	5

***Comment 1***[**:**	6](#_Toc221025270)

***Comment*** [***2:***	7](#_Toc221025271)

***Comment*** [***3:***	7](#_Toc221025272)

***Comment*** [***4:***	8](#_Toc221025273)

***Comment*** [***5:***	9](#_Toc221025274)

**[Reviewer #2’s Comments and Author’s Responses:	9](#_Toc221025280)**

***Comment*** [***1:***	11](#_Toc221025281)

***Comment*** [***2:***	11](#_Toc221025282)

***Comment*** [***3:***	12](#_Toc221025283)

[**Reviewer #3’s Comments and Author’s Responses:	12**](#_Toc221025286)

***Comment*** [***1:***	14](#_Toc221025287)

***Comment*** [***2:***	15](#_Toc221025288)

***Comment*** [***3:***	16](#_Toc221025289)

***Comment*** [***4:***	17](#_Toc221025290)

***Comment*** [***5:***	17](#_Toc221025291)

***Comment*** [***6:***	18](#_Toc221025292)

***Comment*** [***7:***	18](#_Toc221025293)

***Comment*** [***8:***	19](#_Toc221025294)

***Comment*** [***9:***	20](#_Toc221025295)

***Comment*** [***10:***	21](#_Toc221025296)

***Comment*** [***11:***	21](#_Toc221025297)

[**Reviewer #4’s Comments and Author’s Responses**](#_Toc221025298)	**22**

***Comment 1***[**:**](#_Toc221025299)	23

***Comment*** [***2:***](#_Toc221025300)	23

***Comment*** [***3:***](#_Toc221025301)	23

***Comment*** [***4:***](#_Toc221025302)	24

# Editor’s Comment and Author’s Response:

Thank you for your submission. I am including the comments that four Reviewers have provided on your paper, all of whom have made a number of suggestions and criticisms, which I hope you will find useful and constructive. Taken together, I would welcome a revision that accounts for their points, drawing particular attention to those regarding the distinction between altitude-associated effects and breed-specific differences, a point which recurs across multiple Reviewers. As the manuscript is framed around high-altitude hypoxia adaptation, I am in particular agreement with the point that the manuscript should "more explicitly acknowledge that the observed differences may reflect a combination of altitude-associated adaptation, breed-specific genetic background, developmental history, and environmental exposure" and take steps to disambiguate these factors, where it is practical to do so. With regard to Reviewer 4, point 4 (that qPCR may be used as a confirmatory validation "in a large number of samples") presupposes that such a large sample size is available, although I understand it may not; thus, this suggestion may be taken as advisory.

**Response:** We sincerely thank the Editor for the careful evaluation of our manuscript and for the constructive summary of the Reviewers’ comments. We especially appreciate the Editor’s emphasis on the need to distinguish altitude-associated effects from breed-specific differences, which was indeed a recurring concern across multiple Reviewers. In response, we have substantially revised the manuscript to address this issue more explicitly and conservatively throughout the Introduction, Results, Discussion, and Conclusions.

Specifically, we now clarify that the comparison between Diqing Tibetan pig (DT) and Diannan small-eared pig (DSE) represents a natural ecological and breed-level contrast, in which hypobaric hypoxia co-occurs with differences in environmental exposure, husbandry history, growth traits, and genetic background. Accordingly, we have revised the wording throughout the manuscript to avoid attributing all observed molecular differences solely to oxygen concentration. Terms implying direct causality or oxygen-specific adaptation have been replaced, where appropriate, with more cautious expressions such as “altitude-associated”, “breed-associated”, “candidate”, “inferred”, or “may contribute to”. We also added a dedicated limitations paragraph discussing the inability of the current study design to fully disentangle hypoxia-specific effects from other ecological and genetic factors.

In addition, we clarified several methodological aspects highlighted by the Reviewers, including the joint-processing strategy for DT and DSE cells, pig-to-human ortholog mapping procedures, criteria for CellChat interaction inference, and isoform-filtering transparency. We also tempered interpretations related to GO/KEGG enrichment, cell-cycle scoring, and pySCENIC regulon analyses to avoid overstatement of computationally inferred results.

To strengthen experimental support for the transcriptomic findings, we added representative photographs of the two pig breeds (Figure 1A) and performed immunofluorescence validation for EBF3, DBX2, and TCF21 in pituitary tissues (Supplementary Figure S4). Following communication with the Handling Editor, we understand that additional population-scale validation approaches, including selection-signature analyses and qPCR validation in larger cohorts, can be appropriately considered as important future directions rather than required analyses within the current revision scope. We agree that these approaches would further strengthen the conclusions. However, given the limited availability of well-matched pituitary samples from these indigenous pig breeds, especially high-altitude DT pig, such analyses are not feasible in the present study. We have therefore explicitly discussed these points as limitations and future directions in the revised manuscript.

We hope that the revised manuscript adequately addresses the Editor’s and Reviewers’ concerns, and we sincerely appreciate the opportunity to improve our work through this constructive review process.

# Reviewer #1’s Comments and Author’s Responses

In this work, Li et al. presented the results of a comprehensive investigation into the cellular and transcriptomic adaptations of pig to high-altitude hypoxia. The study focuses on porcine pituitary transcriptomes, aiming to understand the mechanisms of hypoxia adaptation in high-altitude environments. This is an important yet underexplored topic, and the complex transcriptional landscape reveals the diverse regulatory mechanisms in two local pig breeds: the Diqing Tibetan pig (DT) and the Diannan small-ear pig (DSE). Additionally, this dataset provides valuable insights for future studies on the gene regulation of high-altitude adaptation. I suggest a minor revision, with a few points for improvement of the manuscript as outlined below.

**Response:** We sincerely thank Reviewer 1 for the positive evaluation of our manuscript and for the constructive suggestions that helped improve its clarity, biological interpretation, and data accessibility. We are grateful for Reviewer 1’s recognition of the importance of investigating pituitary transcriptomic features in high-altitude pig and the potential value of integrating short-read and long-read single-cell sequencing.

In response to the reviewer’s comments, we revised the Introduction to provide more detailed background information on Diqing Tibetan pig (DT) and Diannan small-ear pig (DSE), including their geographic origins, ecological environments, representative phenotypic characteristics, and the need to interpret the comparison in the context of both altitude-associated and breed-associated differences. We also clarified the biological rationale for focusing on the pituitary gland and more explicitly described the knowledge gaps addressed by short-read and long-read single-cell sequencing.

In the Results and Discussion, we expanded the biological interpretation of the long-read isoform findings and the GO/KEGG enrichment results, with particular attention to isoform diversity, mitochondrial energy metabolism, redox regulation, proteostasis, vesicle-mediated transport, and translation-related pathways. We also revised the wording where necessary to maintain a cautious interpretation of altitude-associated pituitary features. All sections revised in response to Reviewer 1’s comments are **highlighted in green** in the revised manuscript.

1) In the introduction section, it may be better to include a bit more details about the two pig breeds. Do they have -any obvious differences other than living at different altitudes?

**Response:** We thank Reviewer 1 for this helpful suggestion. We agree that providing more detailed background information on the two indigenous pig breeds would help readers better understand the biological context of our comparison. In the revised Introduction, we have expanded the description of Diannan small-ear pig (DSE) and Diqing Tibetan pig (DT). Specifically, we now describe their geographic origins and approximate altitudes, with DSE originating from Xishuangbanna (~500 m) and DT mainly distributed around Shangri-La (~3,200 m). We also added details on their contrasting ecological environments and representative phenotypic characteristics. DSE pig are associated with hot and humid lowland environments and are characterized by early maturity, relatively rapid fattening, small body size, and fine bone structure, whereas DT pig inhabit alpine meadow and forest-edge habitats and have been exposed to chronic high-altitude environmental pressures, including hypobaric hypoxia, low temperature, stronger ultraviolet radiation, and seasonal nutritional fluctuation. Phenotypically, DT pig generally show slower growth, stronger environmental tolerance, and distinctive meat-quality traits.

Importantly, we also revised the Introduction to emphasize that the distinct ecological backgrounds and breed-specific characteristics of DT and DSE pigs provide a valuable natural framework for investigating altitude-associated pituitary variation in indigenous pig breeds. This framing helps clarify the biological context of the DT-DSE comparison and avoids overattributing the observed molecular differences to a single environmental factor. **(Line 74-83)**

2) The hypothesis regarding the role of pituitary gland adaptation to high-altitude hypoxia could be stated more explicitly in the introduction. It is important to set the stage more clearly by explaining the specific knowledge gaps that this study aims to address.

**Response:** We appreciate this important and constructive comment. We agree that the central hypothesis and knowledge gap should be stated more explicitly in the Introduction. In the revised Introduction, we have strengthened this section in two main ways. First, we now provide a clearer biological rationale for focusing on the pituitary gland, emphasizing that the pituitary is a central endocrine organ involved in metabolism, growth, stress responses, and reproductive function, all of which may be influenced by chronic altitude-related environmental stress. Based on this rationale, we more explicitly state our working hypothesis that long-term high-altitude environmental pressure may drive pituitary remodeling at the cell-type, transcriptional, intercellular signaling, regulatory, and isoform levels.

Second, we clarified the specific knowledge gap addressed by this study. We now state that how the porcine pituitary adapts to long-term altitude-associated environmental pressure at single-cell resolution remains poorly understood, particularly with respect to changes in cell-type composition, cell-type-specific gene expression, inferred intercellular communication, transcription-factor regulon activity, and transcript isoform usage. We also added a clearer description of how integrating Illumina short-read and Oxford Nanopore long-read single-cell sequencing allowed us to examine both gene-level and isoform-level features in DT and DSE pigs. These revisions now better set the stage for the study and clarify the rationale for our experimental design. **(Line 100-107)** **(Line 137-1****42)**

3) The integration of long-read data and the resulting isoform diversity (~28% novel annotations) is intriguing, and further exploration of how this isoform complexity might influence gene regulation or metabolic pathways in high-altitude adaptation would be valuable. This aspect could be expanded in the discussion.

**Response:** We thank Reviewer 1 for highlighting this important point. We agree that the long-read single-cell data, especially the identification of approximately 28% previously unannotated NIC or NNC isoforms, represent an important aspect of our study and warrant further biological interpretation. In the revised Discussion, we expanded the isoform-related paragraph to better explain the potential regulatory significance of this isoform diversity. Specifically, we now discuss that novel isoforms may influence gene regulation through changes in coding potential, untranslated-region structure, transcript stability, protein-domain composition, and possibly subcellular localization.

We further added that isoform-level regulation may provide an additional regulatory layer beyond gene-level expression, allowing specific pituitary cell populations to fine-tune metabolic, proteostatic, and microenvironmental responses under chronic altitude-associated environmental pressure. We also discuss the observed DT-related isoform patterns, including broader isoform sharing across pituitary cell types and the preferential use of dominant isoforms in non-secretory cells, as potential indicators of coordinated or streamlined transcript regulation. These revisions expand the biological interpretation of the long-read sequencing results while acknowledging that the functional consequences of these isoform changes require further validation. **(Line 582-599)**

4) In the GO and KEGG enrichment analysis results, how do these upregulated pathways contribute to the animals' adaptation and survival in high-altitude environments?

**Response:** We appreciate Reviewer 1’s request for a clearer biological interpretation of the GO and KEGG enrichment results. In response, we revised the Results section to more explicitly explain how these enriched pathways may be related to altitude-associated physiological responses. Specifically, we now explain that enrichment of mitochondrial respiratory chain and oxidative phosphorylation pathways may help maintain ATP production in DT pituitary cells under chronic environmental stress, whereas oxidoreductase-related pathways may contribute to redox homeostasis. We also discuss that vesicle-mediated transport may be important for membrane trafficking, receptor or secretory-vesicle regulation, and intercellular communication, while proteasome-related pathways may support the selective removal of damaged or misfolded proteins caused by hypoxia- and stress-associated oxidative burden.

Conversely, we clarified that the relative downregulation of ribosome- and translation-related pathways may reflect a possible energy-conserving strategy, in which global biosynthetic activity is reduced while essential energy metabolism, stress-response, and protein-quality-control processes are maintained. These revisions provide a clearer biological interpretation of how the enrichment patterns may contribute to pituitary cell function and altitude-associated adaptation in DT pig. **(****Line 259-267)**

5) Make sure the data for this paper has been submit to a public database.

**Response:** We thank Reviewer 1 for this important reminder. We have revised the Data Availability section to clearly state where the sequencing data have been deposited. Specifically, the raw short-read and long-read single-cell sequencing data have been deposited in the NCBI Sequence Read Archive under BioProject accession PRJNA1392118 and in the Genome Sequence Archive under accession CRA035532. We apologize that the datasets were not publicly accessible during the initial review process because the repositories had not yet been released. Upon submission of the revised manuscript, the corresponding datasets have now been made publicly available for access and download.

# Reviewer #2’s Comments and Author’s Responses:

Overall assessment: Structurally and linguistically the manuscript is sound; I have no major objections. It is perhaps somewhat long, but one of its strengths is the use of long-read sequencing and the inclusion of splice-event analysis.

Major concerns

The main weakness of the experimental conclusions is that the two habitats compared differ in many environmental parameters, not only oxygen concentration. Therefore it is not possible to attribute all observed differences specifically to hypoxia.

A further limitation is the low sample size: if I understand correctly, the study was not replicated across multiple individuals/animals, and the analysis is based on comparisons within a very small number of samples.

**Response:** We sincerely thank Reviewer 2 for the careful assessment of our manuscript and for recognizing the strengths of the study, particularly the use of long-read sequencing and splice-event/isoform analysis. We also appreciate Reviewer 2’s important comments regarding the interpretation of hypoxia-related effects, the limitations associated with environmental confounding and small sample size, the use of Guppy for Nanopore basecalling, and the typographical issue in the supplementary figure.

In response, we carefully reviewed the manuscript and revised wording that could imply that the observed differences were caused solely by oxygen availability or hypoxia. We now describe the findings more cautiously as altitude-associated or breed-associated features and explicitly acknowledge in the Discussion and Conclusions that oxygen availability is only one of several ecological differences between the two habitats. We also expanded the limitation statement regarding the small sample size and limited biological replication. In addition, we clarified that Guppy was used because the samples were sequenced and basecalled through the sequencing provider’s validated workflow before Dorado had been incorporated into that workflow, and because the downstream analysis pipeline had been optimized using Guppy-generated FASTQ files. Finally, we corrected the “1 samples” typographical error in the revised Supplementary Figure S5 and checked the manuscript and figure legends for similar inconsistencies. All sections revised in response to Reviewer 2’s comments are **highlighted in yellow** in the revised manuscript.

1) The Nanopore data were basecalled with Guppy to produce FASTQ files. Since Nanopore has recommended Dorado as the preferred basecaller for over a year, is there a specific reason Guppy was used (e.g., timing of sequencing, pipeline constraints), or was Dorado not available at the time?

**Response:** We thank Reviewer 2 for this constructive methodological comment. At the time when the samples were prepared, sequenced, and subjected to primary basecalling by the sequencing provider, Dorado had not yet been incorporated into the provider’s validated production workflow. The Nanopore data were therefore basecalled using Guppy, which was the basecaller implemented in the provider’s standardized workflow at that stage. In addition, the downstream single-cell barcode recovery, read assignment, and isoform-identification pipeline had been established and optimized using Guppy-generated FASTQ files.

We acknowledge that Dorado may provide improved read-level accuracy and could further refine isoform discovery and quantification in future reprocessing. However, because all samples in this study were processed uniformly using the same basecalling and downstream analysis workflow, the comparative isoform-level analyses between DT and DSE pig remain internally consistent.

2) I recommend tempering statements that attribute differences solely to oxygen concentration. Oxygen is only one of several differing environmental factors between the sites, and this should be explicitly acknowledged in the Discussion/Conclusions.

**Response:** We agree with Reviewer 2’s recommendation. In the revised manuscript, we carefully reviewed and revised wording that could be interpreted as attributing all observed differences solely to oxygen availability or hypoxia. Specifically, terms such as “hypoxia-driven,” “mechanisms of high-altitude hypoxia adaptation,” and “directly contribute to hypoxia adaptation” were replaced, where appropriate, with more cautious expressions, including “altitude-associated,” “hypoxia-associated,” “may contribute to,” and “candidate features associated with high-altitude environments.”

We also revised the Discussion and Conclusions to explicitly acknowledge that oxygen availability is only one component of the ecological differences between the two habitats, and that other environmental and breed-related factors may also contribute to the observed molecular differences. These revisions help ensure that our interpretations are more cautious and better aligned with the comparative design of the study. **(Line 44) (Line 51-52) (Line 54-57)** **(Line 114-115) (Line 253) (Line 463-468)** **(Line 510) (Line 613-618)**

3) Minor correction: in S4 Figure change “1 samples” to “1 sample”.

**Response:** We thank Reviewer 2 for pointing out this typographical error. We have corrected “1 samples” to “1 sample” in the revised Supplementary Figure S5, which was formerly Supplementary Figure S4 before the supplementary figures were renumbered. We also carefully checked the figure legends and labels throughout the manuscript for similar grammatical or formatting inconsistencies.

# Reviewer #3’s Comments and Author’s Responses:

In this work, Li et al. present an integrative single-cell transcriptomic study of porcine pituitary tissues from two indigenous pig breeds inhabiting distinct altitudinal environments, namely the high-altitude Diqing Tibetan pig (DT) and the low-altitude Diannan small-ear pig (DSE). By combining short-read single-cell RNA sequencing with Oxford Nanopore-based long-read single-cell sequencing, the authors aim to characterize pituitary cellular composition, transcriptional heterogeneity, cell-cycle states, functional enrichment patterns, cell-cell communication networks, transcription-factor regulon activity, and transcript isoform diversity. The topic is biologically relevant, as the pituitary gland serves as a central endocrine regulatory organ and may participate in systemic responses to chronic hypoxia. The integration of gene-level single-cell profiling with isoform-level long-read information represents a notable strength of the study. Overall, the dataset is valuable, and the analytical framework is relatively comprehensive. However, several issues require clarification before the conclusions can be fully supported, particularly regarding the distinction between altitude-associated effects and breed-specific differences, the interpretation of computationally inferred results, and the transparency of long-read isoform filtering. Therefore, some details still need to be revised, and it is recommended to review the manuscript after revising.

**Response:** We sincerely thank Reviewer 3 for the thorough and insightful evaluation of our manuscript. We are grateful for the recognition of the biological relevance of the porcine pituitary and the value of integrating short-read and long-read single-cell transcriptomics to characterize cell-type heterogeneity, regulatory features, and isoform-level diversity. We also appreciate Reviewer 3’s constructive comments regarding the interpretation of altitude-associated versus breed-associated differences, the need to avoid overinterpreting computationally inferred results, and the importance of improving methodological transparency.

In response to these comments, we revised the Introduction and Discussion to more explicitly acknowledge that the DT-DSE comparison represents a natural ecological and breed-level contrast, in which altitude exposure co-occurs with differences in genetic background, growth traits, husbandry or developmental history, and long-term environmental exposure. We therefore now interpret the observed molecular features more cautiously as altitude-associated signatures in native breed contexts rather than oxygen-specific effects alone. We also clarified the biological rationale for focusing on the pituitary gland and the specific analytical questions addressed by short-read and long-read single-cell sequencing.

We further revised the Results and Methods to improve the transparency and interpretation of the computational analyses. Specifically, we clarified that DT and DSE cells were jointly processed rather than subjected to explicit anchor-based or Harmony batch correction before differential expression analysis; reported the pig-to-human ortholog mapping rate and the handling of unmapped or ambiguous genes; revised the wording for cell-cycle scoring, GO/KEGG enrichment, CellChat communication inference, and pySCENIC regulon analysis to emphasize their inferred nature; and expanded the CellChat and pySCENIC method descriptions, including ligand-receptor filtering, significance criteria, and the rationale for prioritizing candidate transcription factors. We also added immunofluorescence staining for selected candidate TFs to experimentally support the transcriptomic observations.

In addition, we expanded the long-read isoform-filtering section to report the number of isoforms removed at each filtering step and the final high-confidence isoform set. Finally, we carefully checked the manuscript, figures, legends, Methods, and supplementary materials to correct inconsistent abbreviations, terminology, and typographical errors. All sections revised in response to Reviewer 3’s comments are **highlighted in pink** in the revised manuscript.

1) The comparison between Diqing Tibetan pigs and Diannan small-ear pigs involves differences not only in altitude exposure but also in genetic background, growth traits, and possibly husbandry history. Therefore, attributing transcriptional differences solely to high-altitude hypoxia adaptation may be too strong. The Introduction and Discussion should more explicitly acknowledge that the observed differences may reflect a combination of altitude-associated adaptation, breed-specific genetic background, developmental history, and environmental exposure.

**Response:** We thank Reviewer 3 for this important and rigorous comment. We agree that the comparison between DT and DSE pig does not allow altitude-associated effects to be fully separated from breed-specific genetic background, growth traits, developmental or husbandry history, and long-term environmental exposure. To address this concern, we revised both the Introduction and Discussion to make this limitation more explicit.

In the revised Introduction, we now provide additional information on the distinct ecological backgrounds and breed-specific characteristics of DT and DSE pig, and frame these features as the native-breed context for investigating altitude-associated pituitary variation, rather than treating altitude exposure as the only relevant difference between the two breeds. In the revised Discussion, we further clarified that the DT-DSE comparison represents a natural ecological and breed-level contrast, in which hypobaric hypoxia co-occurs with other environmental and biological differences, including temperature, ultraviolet exposure, nutrition, husbandry history, growth traits, and genetic background. Accordingly, we now interpret the molecular features identified in this study as high-altitude-associated signatures in native breed contexts, rather than as oxygen-specific effects alone.

We also added a limitation paragraph emphasizing that future studies with larger cohorts, controlled or reciprocal environmental designs, physiological measurements, population-level selection-signature analyses, and additional molecular validation will be needed to distinguish oxygen-specific effects from other ecological, developmental, and genetic influences. These revisions make the interpretation more cautious and better aligned with the comparative design of the study. **(Line 83-85) (Line 600-610)**

2) In the Introduction, the pituitary is described as an endocrine hub, but the central hypothesis is not sufficiently explicit. The authors should clarify why pituitary remodeling is expected under high-altitude hypoxia and what specific questions are addressed by short-read and long-read single-cell sequencing.

**Response:** We appreciate this helpful suggestion. In the revised Introduction, we have more clearly explained the biological rationale for focusing on the pituitary and the analytical questions addressed in this study. We now state that, as a central endocrine organ coordinating metabolism, growth, stress responses, and reproductive function, the pituitary may be sensitive to long-term altitude-associated environmental stress, including hypobaric hypoxia, because systemic adaptation requires coordinated endocrine regulation. We therefore reasoned that altitude-associated environmental pressure may be reflected in pituitary cell composition, cell-type-specific transcriptional programs, intercellular signaling, and transcript isoform structure.

We also clarified the specific questions addressed by each sequencing platform. Short-read single-cell sequencing was used to resolve cell-type composition, cell-type-specific gene expression, inferred cell-cycle states, and intercellular signaling patterns, whereas long-read single-cell sequencing was used to reconstruct full-length isoforms and characterize transcript structural diversity. Together, these revisions make the biological rationale and analytical logic of the study more explicit. **(Line 127-137)**

3) Cells from DT and DSE pituitaries appear to be integrated before clustering and downstream comparison. However, integration or batch correction may remove true biological differences between breeds, whereas insufficient correction may preserve technical variation. The authors should clarify which integration method was used, which variables were corrected, and whether key findings were robust before and after integration.

**Response:** We thank Reviewer 3 for raising this important methodological concern. We agree that integration or batch correction may potentially attenuate true biological differences between breeds, whereas insufficient correction may allow technical variation to persist. In the revised manuscript, we have clarified our data-processing strategy to avoid ambiguity.

Specifically, DT and DSE cells were merged and jointly processed using the same Seurat workflow, including normalization, highly variable feature selection, PCA, UMAP visualization, nearest-neighbor graph construction, clustering, and cell-type annotation. Importantly, we did not apply anchor-based integration, Harmony, or other explicit batch-correction methods before differential expression analysis, in order to avoid removing breed- or altitude-associated biological variation. We have also replaced the ambiguous term “data integration” with “joint processing” where appropriate. **(Line 151-152)**

4) Several analyses depend on mapping porcine genes to human homologs, including cell-type annotation, ligand–receptor inference, and regulatory network analysis. However, incomplete or many-to-one ortholog mapping may result in gene loss or incorrect functional assignment. The authors should report the mapping rate and discuss how unmapped or ambiguously mapped genes were handled.

**Response:** We thank Reviewer 3 for highlighting this important methodological point. We agree that incomplete or ambiguous pig-to-human ortholog conversion may lead to gene loss or potential functional misassignment in human-reference-dependent analyses, including cell-type annotation, ligand-receptor inference, and regulon analysis. In the revised manuscript, we therefore reported the pig-to-human ortholog mapping rate and clarified how unmapped or ambiguously mapped genes were handled.

Specifically, porcine genes were converted to human orthologs using Ensembl BioMart, and only confidently assigned orthologs were retained for downstream analyses requiring human gene annotations. The final confident mapping rate was 14,985 of 28,932 porcine genes (51.79%). Genes without mapped human orthologs were excluded from human-reference-dependent analyses. For genes with many-to-one or one-to-many ortholog relationships, we retained the best-supported ortholog only when a clear assignment was available based on orthology confidence and sequence similarity; otherwise, these genes were excluded to minimize incorrect functional assignment. Although this filtering may reduce gene coverage, it provides a more conservative framework for cross-species annotation and downstream inference. **(Line732-740)**

5) Cell-cycle scoring is used to compare proliferative states between DT and DSE pituitary cells. However, cell-cycle phase assignment based on transcriptomic signatures is computational inference and should not be interpreted as direct evidence of cell proliferation. The authors should revise the wording to avoid overstatement.

**Response:** We thank Reviewer 3 for this constructive and important comment. We agree that cell-cycle phase assignment based on transcriptomic signatures is a computational inference and should not be interpreted as direct experimental evidence of cell proliferation. In the revised manuscript, we have carefully revised the relevant wording in the Results, figure legend, and Discussion to avoid overstatement.

Specifically, phrases such as “elevated proliferative activity,” “cell cycle arrest,” and “suppressed proliferation” have been replaced with more cautious descriptions, including “higher transcriptomically inferred S/G2M-phase signature,” “G1-biased cell-cycle signature,” and “higher inferred S/G2M-phase fraction.” These revisions clarify that the analysis reflects inferred cell-cycle-associated transcriptional states rather than directly measured proliferation. **(Line 184-188)** **(Line 205-206)**

6) GO/KEGG enrichment is interpreted as evidence for metabolic reprogramming and adaptation. However, enrichment analysis is computational inference rather than direct functional validation. The authors should avoid using phrases that imply functional testing and should clarify that these pathways represent predicted or inferred biological processes.

**Response:** We appreciate this important comment. We agree that GO and KEGG enrichment analyses provide computationally inferred functional associations based on differentially expressed genes, rather than direct experimental validation of pathway activity or biological function. To avoid overstatement, we revised the wording in the Results section to use more cautious language. Specifically, we replaced wording that could imply direct functional evidence with “suggested” when describing GO and KEGG enrichment patterns. These revisions clarify that the enriched terms and pathways should be interpreted as predicted biological associations based on gene-set enrichment, rather than experimentally validated mechanisms of adaptation. **(Line246-247)**

7) DSE is reported to show greater overall communication quantity and strength, whereas DT shows stronger communication in non-hormone-secreting populations. This pattern is interesting but insufficiently explained. The authors should clarify how these results can coexist and what they imply biologically.

**Response:** We thank Reviewer 3 for pointing out the need for a clearer explanation. In the revised Results, we clarified that these two observations describe different levels of the inferred communication network and are therefore not contradictory. The greater total communication quantity and strength in DSE reflect aggregate signaling across all annotated cell types, which may be influenced by the larger contribution of hormone-secreting populations and broadly distributed endocrine-related interactions. In contrast, the stronger communication observed in DT non-hormone-secreting populations indicates a more localized enhancement of stromal, vascular, endothelial, immune, and pericyte-associated signaling.

Biologically, these results suggest that DSE may retain a globally more active endocrine communication network, whereas DT may concentrate signaling activity within non-secretory microenvironmental niches potentially related to tissue maintenance, vascular remodeling, and local stress responses. These revisions clarify how the two findings can coexist and provide a more cautious interpretation of their biological implications. **(Line277-285)**

8) The study identifies 20 differential ligand–receptor axes, including collagen–integrin, PECAM1–PECAM1, NCAM1–NCAM2, and PTN–SDC2. However, it is unclear how significance was determined, whether expression thresholds were applied, and how pig-to-human ortholog mapping affected the analysis.

**Response:** We thank Reviewer 3 for this constructive methodological suggestion. We agree that the criteria used to identify differential ligand-receptor interactions should be described more transparently. In the revised Methods, we expanded the CellChat analysis section to clarify the pig-to-human ortholog mapping procedure, the filtering of ligand-receptor pairs, and the statistical criteria used to define significant interactions.

Specifically, porcine genes were first converted to human orthologs before matching to the CellChat human ligand-receptor database, and only ligand-receptor pairs for which both ligand and receptor genes were successfully mapped were retained. Unmapped or ambiguously mapped genes were excluded from this human-reference-dependent analysis. We also clarified that overexpressed ligands and receptors were identified using CellChat’s default expression-filtering and statistical framework, with no additional manual expression threshold applied. Communication probabilities were inferred using permutation-based testing, and differential ligand-receptor pairs between DT and DSE were ranked according to the absolute difference in inferred communication probability. Pairs with adjusted P-value < 0.05 were considered significant. **(Line732-740)**

9) EBF3, DBX2, TCF21, TEAD4, LHX3, and RUNX3 are nominated as potential regulators of pituitary hypoxia adaptation. However, pySCENIC infers regulon activity from co-expression and motif enrichment; it does not prove direct regulatory function. The authors should avoid presenting these TFs as validated drivers and should explain why these specific TFs are biologically relevant.

**Response:** We fully agree with Reviewer 3. pySCENIC identifies candidate regulons based on co-expression and motif enrichment, and we have therefore revised the wording to avoid presenting EBF3, DBX2, TCF21, TEAD4, LHX3, and RUNX3 as confirmed drivers of pituitary adaptation. In the revised manuscript, these factors are now described more cautiously as candidate regulators or inferred regulons associated with altitude-related pituitary variation.

We also clarified the rationale for prioritizing these TFs. They were highlighted because they showed the highest RSS values in their corresponding cell types, indicating strong cell-type specificity of inferred regulon activity. Given the biological roles of the corresponding cell types, these regulon patterns may provide clues to transcriptional programs associated with pituitary cell identity, tissue remodeling, and microenvironmental regulation under altitude-associated conditions. In addition, immunofluorescence staining for selected TFs, including TCF21, DBX2, and EBF3, provided protein-level support for their expression in porcine pituitary tissues. Thus, we present these TFs as prioritized candidate regulators supported by cell-type-specific regulon inference and, for selected factors, protein-level staining evidence. **(Line340-346)**

10) The study states that isoforms with non-canonical splice junctions, NMD prediction, or 3′ terminal deviations greater than 100 bp were excluded. However, the exact number of transcripts removed at each filtering step is not provided.

**Response:** We thank Reviewer 3 for pointing out this missing methodological detail. In the revised Methods, we expanded the isoform classification and filtering section to report the number of isoforms removed at each filtering step. Specifically, we now describe the sequential filtering procedure starting from 110,214 initially classified isoforms, followed by removal of isoforms with non-canonical splice junctions, isoforms flagged at the RTS-filtering step, predicted NMD targets, and isoforms with 3′ terminal deviations greater than or equal to 100 bp from annotated polyadenylation sites. We also report the final number of isoforms retained after filtering. This revision provides a clearer stepwise description of how the high-confidence isoform catalogue was generated and how each filtering criterion contributed to the final isoform set. **(Line787-795)**

11) There are inconsistencies in the manuscript, including DT/DSE versus DT/DST or DES, “DGEs” versus “DEGs,” “BLZAE” versus “BLAZE,” and spelling errors such as “intergrative,” “denonstrated,” and “hypoxi-driven.” These issues reduce readability and should be corrected carefully.

**Response:** We apologize for these inconsistencies and thank Reviewer 3 for the careful reading. We have thoroughly checked the manuscript, figures, figure legends, Methods, and supplementary materials to correct inconsistent abbreviations and typographical errors. Specifically, instances of “DT/DST” and “DES” were corrected to “DT/DSE” where appropriate, “DGEs” was corrected to “DEGs,” “BLZAE” was corrected to “BLAZE,” and spelling errors such as “intergrative,” “denonstrated,” and “hypoxi-driven” were corrected.We also reviewed the figure legends and supplementary materials to ensure consistency in cell-type names, breed abbreviations, and isoform-category terminology. These corrections improve the readability, accuracy, and consistency of the revised manuscript. **(Line 95)** **(Line 162) (Line 170-171) (Line 240) (Line 315) (Line 370)** **(Line 384) (Line 422)** **(Line 432) (Line 459)**

# Reviewer #4’s Comments and Author’s Responses:

The authors have used short-read and long-read single-cell transcriptomics of pig pituitary to decipher mechanisms of high-altitude hypoxia adaptation. however, few cooments need to be addressed before final acceptance.

**Response:** We sincerely thank Reviewer 4 for the positive evaluation of our work and for recognizing the value of integrating short-read and long-read single-cell transcriptomics to investigate altitude-associated pituitary features in pig. We appreciate Reviewer 4’s careful reading of the manuscript and the constructive suggestions for improving the experimental support and presentation of the study.

In response to Reviewer 4’s comments, we first replaced the schematic black pig illustrations in the technical workflow of Fig 1A with high-quality representative photographs of Diqing Tibetan pig (DT) and Diannan small-eared pig (DSE), providing a clearer visual presentation of the two indigenous pig breeds. We also performed immunofluorescence staining for EBF3, DBX2, and TCF21 in porcine pituitary tissues and added representative images to Supplementary Figure S4, thereby experimentally validating the transcriptomic observations for selected candidate regulatory genes.

For the suggestions regarding selection-signature analysis and qPCR validation in larger cohorts, we sought guidance from the Handling Editor. Following the Editor’s guidance, we understand that these analyses can be appropriately addressed as important future directions rather than required analyses within the current revision scope. We have therefore revised the Discussion to explicitly acknowledge that future studies integrating population genomics, larger-cohort validation, and single-cell transcriptomics will be needed to further strengthen and extend the present findings. All sections revised in response to Reviewer 4’s comments are **highlighted in blue** in the revised manuscript.

1) Kindly include good quality photographs of both Diqing Tibetan pig and Diannan small-eared pig.

**Response:** We thank Reviewer 4 for this helpful suggestion. In response, we replaced the schematic black pig illustrations in the technical workflow of Fig 1A with high-quality representative photographs of Diqing Tibetan pig (DT) and Diannan small-eared pig (DSE). These photographs provide a clearer and more realistic visual presentation of the two indigenous pig breeds, allowing readers to better appreciate their phenotypic characteristics and ecological backgrounds.

2) EBF3, DBX2, and TCF21 genes need to be functionally validated.

**Response:** We appreciate Reviewer 4’s important comment regarding experimental validation of the candidate regulatory genes. In response, we performed immunofluorescence staining for EBF3, DBX2, and TCF21 in porcine pituitary tissues and added representative images to Supplementary Figure S4. These results provide experimental validation of the transcriptomic observations for selected candidate regulatory genes and are consistent with the cell-type-associated expression patterns inferred from the single-cell transcriptomic and regulon analyses. The corresponding figure legend has also been revised accordingly. **(Line 346-350)**

3) Selection signatures analysis is strongly recommended to validate the findings.

**Response:** We thank Reviewer 4 for this valuable suggestion. We agree that selection-signature analysis would provide useful population-genomic evidence for understanding altitude-associated adaptation. We also sought guidance from the Handling Editor regarding this point. Following the Editor’s guidance, we understand that this suggestion can be considered an important future direction, as it would require population-scale genomic data that are not part of the current single-cell transcriptomic dataset.

A rigorous selection-signature analysis would generally require whole-genome resequencing or genome-wide SNP data from a sufficient number of well-defined and unrelated individuals from both high- and low-altitude populations. In contrast, the present study was designed to characterize pituitary cell-type-specific gene expression and isoform-level variation using short-read and long-read single-cell transcriptomic data. Therefore, while we fully recognize the value of selection-signature analysis, we have treated this analysis as an important future direction rather than adding it to the current revision.

4) qPCR may also be used to validate the study in large number of samples.

**Response:** We thank Reviewer 4 for this helpful suggestion. We agree that qPCR validation in a larger independent sample set would further strengthen the study. We also sought guidance from the Handling Editor regarding the expected level of validation in this revision. Following the Editor’s guidance, we understand that the request for validation can be appropriately addressed by immunofluorescence validation to experimentally confirm the transcriptome-based observations within the pituitary.

Accordingly, we performed immunofluorescence staining for selected candidate transcription factors, including EBF3, DBX2, and TCF21, in porcine pituitary tissues, and added representative images to the revised Supplementary Figure S4. These immunofluorescence results experimentally validate the transcriptomic observations for selected candidate regulatory genes and support the cell-type-associated expression patterns inferred from the single-cell transcriptomic analyses. Given the limited availability of well-matched pituitary samples from these indigenous pig breeds, especially high-altitude DT pigs, we have treated larger-cohort qPCR validation as an important future direction rather than an additional analysis within the current revision.
