# Supplementary material for: Integrating short-read and long-read single-cell transcriptomics of pig pituitary reveals mechanisms of high-altitude hypoxia adaptation
Source: PLoS Genet. 2026 Aug 10;22(8):e1012267. doi: 10.1371/journal.pgen.1012267 (PMC13472502; doi:10.1371/journal.pgen.1012267)
Supplement: Peer review history

# Peer review: Decision Letter 0

**DOI:** 10.1371/journal.pgen.1012267.r001

**Authors:** Giorgio Sirugo, Stephen Bush

9 May 2026

PGENETICS-D-26-00113

Integrating short-read and long-read single-cell transcriptomics of pig pituitary reveals mechanisms of high-altitude hypoxia adaptation

PLOS Genetics

Dear Dr. Huo,

Thank you for submitting your manuscript to PLOS Genetics. After careful consideration, we feel that it has merit but does not fully meet PLOS Genetics's publication criteria as it currently stands. Therefore, we invite you to submit a revised version of the manuscript that addresses the points raised during the review process.

Please submit your revised manuscript by Jul 08 2026 11:59PM. If you will need more time than this to complete your revisions, please reply to this message or contact the journal office at plosgenetics@plos.org. When you're ready to submit your revision, log on to https://www.editorialmanager.com/pgenetics/ and select the 'Submissions Needing Revision' folder to locate your manuscript file.

Please include the following items when submitting your revised manuscript:

* A letter that responds to each point raised by the editor and reviewer(s). You should upload this letter as a separate file labeled 'Response to Reviewers'. This file does not need to include responses to any formatting updates and technical items listed in the 'Journal Requirements' section below.

* A marked-up copy of your manuscript that highlights changes made to the original version. You should upload this as a separate file labeled 'Revised Manuscript with Track Changes'.

* An unmarked version of your revised paper without tracked changes. You should upload this as a separate file labeled 'Manuscript'.

If you would like to make changes to your financial disclosure, competing interests statement, or data availability statement, please make these updates within the submission form at the time of resubmission. Guidelines for resubmitting your figure files are available below the reviewer comments at the end of this letter.

As the corresponding author, your ORCID iD is verified in the submission system and will appear in the published article. PLOS supports the use of ORCID, and we encourage all coauthors to register for an ORCID iD and use it as well. Please encourage your coauthors to verify their ORCID iD within the submission system before final acceptance, as unverified ORCID iDs will not appear in the published article. Only the individual author can complete the verification step; PLOS staff cannot verify ORCID iDs on behalf of authors.

We look forward to receiving your revised manuscript.

Kind regards,

Stephen Bush

Academic Editor

PLOS Genetics

Giorgio Sirugo

Section Editor

PLOS Genetics

Aimée Dudley

Editor-in-Chief

PLOS Genetics

Anne Goriely

Editor-in-Chief

PLOS Genetics

**Additional Editor Comments:**

Thank you for your submission. I am including the comments that four reviewers have provided on your paper, all of whom have made a number of suggestions and criticisms, which I hope you will find useful and constructive. Taken together, I would welcome a revision that accounts for their points, drawing particular attention to those regarding the distinction between altitude-associated effects and breed-specific differences, a point which recurs across multiple reviewers. As the manuscript is framed around high-altitude hypoxia adaptation, I am in particular agreement with the point that the manuscript should "more explicitly acknowledge that the observed differences may reflect a combination of altitude-associated adaptation, breed-specific genetic background, developmental history, and environmental exposure" and take steps to disambiguate these factors, where it is practical to do so. With regard to reviewer 4, point 4 (that qPCR may be used as a confirmatory validation "in a large number of samples") presupposes that such a large sample size is available, although I understand it may not; thus, this suggestion may be taken as advisory.

**Journal Requirements:**

1) Please ensure that the CRediT author contributions listed for every co-author are completed accurately and in full.

At this stage, the follwing Authors/Authors require contributions: Hai Li, Xudong Wu, Hailong Huo, Xia Zhang, Yiming Guo, Wan Lin, Feidi Wen, Zhijun Zhao, Guiying Zhao, and Jinlong Huo. Please ensure that the full contributions of each author are acknowledged in the "Add/Edit/Remove Authors" section of our submission form.

The list of CRediT author contributions may be found here: https://journals.plos.org/plosgenetics/s/authorship#loc-author-contributions

2) Please upload all main figures as separate Figure files in .tif or .eps format. For more information about how to convert and format your figure files please see our guidelines:

https://journals.plos.org/plosgenetics/s/figures

3) Some material included in your submission may be copyrighted. According to PLOS's copyright policy, authors who use figures or other material (e.g., graphics, clipart, maps) from another author or copyright holder must demonstrate or obtain permission to publish this material under the Creative Commons Attribution 4.0 International (CC BY 4.0) License used by PLOS journals. Please closely review the details of PLOS's copyright requirements here: <a data-cke-saved-href="https://journals.plos.org/plosgenetics/s/licenses-and-copyright" href="https://journals.plos.org/plosgenetics/s/licenses-and-copyright">PLOS Licenses and Copyright</a>. If you need to request permissions from a copyright holder, you may use the&nbsp;<a data-cke-saved-href="https://storage.googleapis.com/plos-published-prod/c4ff/Content%20Copyright%20Permission%20Form.pdf?X-Goog-Algorithm=GOOG4-RSA-SHA256&X-Goog-Credential=wombat-sa%40plos-prod.iam.gserviceaccount.com%2F20220817%2Fauto%2Fstorage%2Fgoog4_request&X-Goog-Date=20220817T210608Z&X-Goog-Expires=86400&X-Goog-SignedHeaders=host&X-Goog-Signature=cd9dc868773378f7033b9f8cfb91430167d6275df90bce01342a454f36512530b64f24736fbf68c4fd96da74d663b6ee95b3420bded8fb0e833af2390b896b8706643b476d7f532408921b60576d65b60617070900fd2bcd28eec4cd32fefcf007d3655df17fcc91023329a28479ce0d1c5758174eedcc9e3c1788c5f7dd8523f3ddd871e02c998a20f7dc78ec00f313d4b5a78ba2037a6c7ef0a05dc29817da9368065460c8d8446659f35f0458fefabd3101eb7f926e9cb7a0b53e7bbe47aaed3e0ba52f9af9c2b8f6d7d3a8871b205fc4cbe97384ed37c7604fd7eecfedccf49cba12820b2e3b649541cefde3103cd7d72731b3e8b0248439b4ba691ca8a" href="https://storage.googleapis.com/plos-published-prod/c4ff/Content%20Copyright%20Permission%20Form.pdf?X-Goog-Algorithm=GOOG4-RSA-SHA256&X-Goog-Credential=wombat-sa%40plos-prod.iam.gserviceaccount.com%2F20220817%2Fauto%2Fstorage%2Fgoog4_request&X-Goog-Date=20220817T210608Z&X-Goog-Expires=86400&X-Goog-SignedHeaders=host&X-Goog-Signature=cd9dc868773378f7033b9f8cfb91430167d6275df90bce01342a454f36512530b64f24736fbf68c4fd96da74d663b6ee95b3420bded8fb0e833af2390b896b8706643b476d7f532408921b60576d65b60617070900fd2bcd28eec4cd32fefcf007d3655df17fcc91023329a28479ce0d1c5758174eedcc9e3c1788c5f7dd8523f3ddd871e02c998a20f7dc78ec00f313d4b5a78ba2037a6c7ef0a05dc29817da9368065460c8d8446659f35f0458fefabd3101eb7f926e9cb7a0b53e7bbe47aaed3e0ba52f9af9c2b8f6d7d3a8871b205fc4cbe97384ed37c7604fd7eecfedccf49cba12820b2e3b649541cefde3103cd7d72731b3e8b0248439b4cba691ca8a">PLOS Content Copyright Permission&nbsp;Form</a>

Please respond directly to this email and provide any known details concerning your material's license terms and permissions required for reuse, even if you have not yet obtained copyright permissions or are unsure of your material's copyright compatibility. Once you have responded and addressed all other outstanding technical requirements, you may resubmit your manuscript within Editorial Manager.

Potential Copyright Issues:

i) Figure 1..: Please confirm whether you drew the images / clip-art within the figure panels by hand. If you did not draw the images, please provide (a) a link to the source of the images or icons and their license / terms of use; or (b) written permission from the copyright holder to publish the images or icons under our CC BY 4.0 license. Alternatively, you may replace the images with open source alternatives. See these open source resources you may use to replace images / clip-art:

- https://commons.wikimedia.org

- https://openclipart.org/

4) Please amend your detailed Financial Disclosure statement. This is published with the article. It must therefore be completed in full sentences and contain the exact wording you wish to be published.

1) State the initials, alongside each funding source, of each author to receive each grant. For example: "This work was supported by the National Institutes of Health (####### to AM; ###### to CJ) and the National Science Foundation (###### to AM)."

2) State what role the funders took in the study. If the funders had no role in your study, please state: "The funders had no role in study design, data collection and analysis, decision to publish, or preparation of the manuscript.".

If you did not receive any funding for this study, please simply state: u201cThe authors received no specific funding for this work.u201d

**Reviewers' comments:**

Reviewer's Responses to Questions

**Comments to the Authors:**

**Please note here if the review is uploaded as an attachment.**

Reviewer #1: In this work, Li et al. presented the results of a comprehensive investigation into the cellular and transcriptomic adaptations of pigs to high-altitude hypoxia. The study focuses on porcine pituitary transcriptomes, aiming to understand the mechanisms of hypoxia adaptation in high-altitude environments. This is an important yet underexplored topic, and the complex transcriptional landscape reveals the diverse regulatory mechanisms in two local pig breeds: the Diqing Tibetan pig (DT) and the Diannan small-ear pig (DSE). Additionally, this dataset provides valuable insights for future studies on the gene regulation of high-altitude adaptation. I suggest a minor revision, with a few points for improvement of the manuscript as outlined below.

(1) In the introduction section, it may be better to include a bit more details about the two pig breeds. Do they have any obvious differences other than living at different altitudes?

(2) The hypothesis regarding the role of pituitary gland adaptation to high-altitude hypoxia could be stated more explicitly in the introduction. It is important to set the stage more clearly by explaining the specific knowledge gaps that this study aims to address.

(3) The integration of long-read data and the resulting isoform diversity (~28% novel annotations) is intriguing, and further exploration of how this isoform complexity might influence gene regulation or metabolic pathways in high-altitude adaptation would be valuable. This aspect could be expanded in the discussion.

(4) In the GO and KEGG enrichment analysis results, how do these upregulated pathways contribute to the animals' adaptation and survival in high-altitude environments?

(5) Make sure the data for this paper has been submit to a public database.

Reviewer #2: Overall assessment: Structurally and linguistically the manuscript is sound; I have no major objections. It is perhaps somewhat long, but one of its strengths is the use of long-read sequencing and the inclusion of splice-event analysis.

Major concerns

The main weakness of the experimental conclusions is that the two habitats compared differ in many environmental parameters, not only oxygen concentration. Therefore it is not possible to attribute all observed differences specifically to hypoxia.

A further limitation is the low sample size: if I understand correctly, the study was not replicated across multiple individuals/animals, and the analysis is based on comparisons within a very small number of samples.

Specific comments / Questions

1, The Nanopore data were basecalled with Guppy to produce FASTQ files. Since Nanopore has recommended Dorado as the preferred basecaller for over a year, is there a specific reason Guppy was used (e.g., timing of sequencing, pipeline constraints), or was Dorado not available at the time?

2, I recommend tempering statements that attribute differences solely to oxygen concentration. Oxygen is only one of several differing environmental factors between the sites, and this should be explicitly acknowledged in the Discussion/Conclusions.

3, Minor correction: in S4 Figure change “1 samples” to “1 sample”.

Reviewer #3: In this work, Li et al. present an integrative single-cell transcriptomic study of porcine pituitary tissues from two indigenous pig breeds inhabiting distinct altitudinal environments, namely the high-altitude Diqing Tibetan pig (DT) and the low-altitude Diannan small-ear pig (DSE). By combining short-read single-cell RNA sequencing with Oxford Nanopore-based long-read single-cell sequencing, the authors aim to characterize pituitary cellular composition, transcriptional heterogeneity, cell-cycle states, functional enrichment patterns, cell-cell communication networks, transcription-factor regulon activity, and transcript isoform diversity. The topic is biologically relevant, as the pituitary gland serves as a central endocrine regulatory organ and may participate in systemic responses to chronic hypoxia. The integration of gene-level single-cell profiling with isoform-level long-read information represents a notable strength of the study. Overall, the dataset is valuable, and the analytical framework is relatively comprehensive. However, several issues require clarification before the conclusions can be fully supported, particularly regarding the distinction between altitude-associated effects and breed-specific differences, the interpretation of computationally inferred results, and the transparency of long-read isoform filtering. Therefore, some details still need to be revised, and it is recommended to review the manuscript after revising.

1. The comparison between Diqing Tibetan pigs and Diannan small-ear pigs involves differences not only in altitude exposure but also in genetic background, growth traits, and possibly husbandry history. Therefore, attributing transcriptional differences solely to high-altitude hypoxia adaptation may be too strong. The Introduction and Discussion should more explicitly acknowledge that the observed differences may reflect a combination of altitude-associated adaptation, breed-specific genetic background, developmental history, and environmental exposure.

2. In the Introduction, the pituitary is described as an endocrine hub, but the central hypothesis is not sufficiently explicit. The authors should clarify why pituitary remodeling is expected under high-altitude hypoxia and what specific questions are addressed by short-read and long-read single-cell sequencing.

3. Cells from DT and DSE pituitaries appear to be integrated before clustering and downstream comparison. However, integration or batch correction may remove true biological differences between breeds, whereas insufficient correction may preserve technical variation. The authors should clarify which integration method was used, which variables were corrected, and whether key findings were robust before and after integration.

4. Several analyses depend on mapping porcine genes to human homologs, including cell-type annotation, ligand–receptor inference, and regulatory network analysis. However, incomplete or many-to-one ortholog mapping may result in gene loss or incorrect functional assignment. The authors should report the mapping rate and discuss how unmapped or ambiguously mapped genes were handled.

5 Cell-cycle scoring is used to compare proliferative states between DT and DSE pituitary cells. However, cell-cycle phase assignment based on transcriptomic signatures is computational inference and should not be interpreted as direct evidence of cell proliferation. The authors should revise the wording to avoid overstatement.

6. GO/KEGG enrichment is interpreted as evidence for metabolic reprogramming and adaptation. However, enrichment analysis is computational inference rather than direct functional validation. The authors should avoid using phrases that imply functional testing and should clarify that these pathways represent predicted or inferred biological processes.

7. DSE is reported to show greater overall communication quantity and strength, whereas DT shows stronger communication in non-hormone-secreting populations. This pattern is interesting but insufficiently explained. The authors should clarify how these results can coexist and what they imply biologically.

8. The study identifies 20 differential ligand–receptor axes, including collagen–integrin, PECAM1–PECAM1, NCAM1–NCAM2, and PTN–SDC2. However, it is unclear how significance was determined, whether expression thresholds were applied, and how pig-to-human ortholog mapping affected the analysis.

9. EBF3, DBX2, TCF21, TEAD4, LHX3, and RUNX3 are nominated as potential regulators of pituitary hypoxia adaptation. However, pySCENIC infers regulon activity from co-expression and motif enrichment; it does not prove direct regulatory function. The authors should avoid presenting these TFs as validated drivers and should explain why these specific TFs are biologically relevant.

10. The study states that isoforms with non-canonical splice junctions, NMD prediction, or 3′ terminal deviations greater than 100 bp were excluded. However, the exact number of transcripts removed at each filtering step is not provided.

11. There are inconsistencies in the manuscript, including DT/DSE versus DT/DST or DES, “DGEs” versus “DEGs,” “BLZAE” versus “BLAZE,” and spelling errors such as “intergrative,” “denonstrated,” and “hypoxi-driven.” These issues reduce readability and should be corrected carefully.

Reviewer #4: The authors have used short-read and long-read single-cell transcriptomics of pig pituitary to decipher mechanisms of high-altitude hypoxia adaptation. however, few cooments need to be addressed before final acceptance.

1. Kindly include good quality photographs of both Diqing Tibetan pig and Diannan small-eared pig.

2. EBF3, DBX2, and TCF21 genes need to be functionally validated.

3. Selection signatures analysis is strongly recommended to validate the findings.

4. qPCR may also be used to validate the study in large number of samples.

**********

**Have all data underlying the figures and results presented in the manuscript been provided?**

Large-scale datasets should be made available via a public repository as described in the *PLOS Genetics*  data availability policy, and numerical data that underlies graphs or summary statistics should be provided in spreadsheet form as supporting information.

Reviewer #1: Yes

Reviewer #2: Yes

Reviewer #3: Yes

Reviewer #4: Yes

**********

PLOS authors have the option to publish the peer review history of their article (what does this mean?). If published, this will include your full peer review and any attached files.

If you choose “no”, your identity will remain anonymous but your review may still be made public.

**Do you want your identity to be public for this peer review?**  For information about this choice, including consent withdrawal, please see our Privacy Policy.

Reviewer #1: No

Reviewer #2: No

Reviewer #3: No

Reviewer #4: No

[NOTE: If reviewer comments were submitted as an attachment file, they will be attached to this email and accessible via the submission site. Please log into your account, locate the manuscript record, and check for the action link "View Attachments". If this link does not appear, there are no attachment files.]

**Figure resubmission:**

After uploading your figures to PLOS’s NAAS tool - https://ngplosjournals.pagemajik.ai/artanalysis, NAAS will process the files provided and display the results in the "Uploaded Files" section of the page as the processing is complete. If the uploaded figures meet our requirements (or NAAS is able to fix the files to meet our requirements), the figure will be marked as "fixed" above. If NAAS is unable to fix the files, a red "failed" label will appear above. When NAAS has confirmed that the figure files meet our requirements, please download the file via the download option, and include these NAAS processed figure files when submitting your revised manuscript.

**Reproducibility:**

To enhance the reproducibility of your results, we recommend that authors of applicable studies deposit laboratory protocols in protocols.io, where a protocol can be assigned its own identifier (DOI) such that it can be cited independently in the future. Additionally, PLOS ONE offers an option to publish peer-reviewed clinical study protocols. Read more information on sharing protocols at https://plos.org/protocols?utm_medium=editorial-email&utm_source=authorletters&utm_campaign=protocols

# Author comment: Author response to Decision Letter 1

**DOI:** 10.1371/journal.pgen.1012267.r002

10 Jun 2026

Attachment Submitted filename: Response to Reviewers.docx

# Peer review: Decision Letter 1

**DOI:** 10.1371/journal.pgen.1012267.r003

**Authors:** Giorgio Sirugo, Stephen Bush

23 Jul 2026

Dear Dr Huo,

We are pleased to inform you that your manuscript entitled "Integrating short-read and long-read single-cell transcriptomics of pig pituitary reveals mechanisms of high-altitude hypoxia adaptation" has been editorially accepted for publication in PLOS Genetics. Congratulations!

Before your submission can be formally accepted and sent to production you will need to complete our formatting changes, which you will receive in a follow up email. Please be aware that it may take several days for you to receive this email; during this time no action is required by you. Please note: the accept date on your published article will reflect the date of this provisional acceptance, but your manuscript will not be scheduled for publication until the required changes have been made.

Once your paper is formally accepted, an uncorrected proof of your manuscript will be published online ahead of the final version, unless you’ve already opted out via the online submission form. If, for any reason, you do not want an earlier version of your manuscript published online or are unsure if you have already indicated as such, please let the journal staff know immediately at plosgenetics@plos.org.

In the meantime, please log into Editorial Manager at https://www.editorialmanager.com/pgenetics/, click the "Update My Information" link at the top of the page, and update your user information to ensure an efficient production and billing process. Note that PLOS requires an ORCID iD for all corresponding authors. Therefore, please ensure that you have an ORCID iD and that it is validated in Editorial Manager. To do this, go to ‘Update my Information’ (in the upper left-hand corner of the main menu), and click on the Fetch/Validate link next to the ORCID field.  This will take you to the ORCID site and allow you to create a new iD or authenticate a pre-existing iD in Editorial Manager.

If you have a press-related query, or would like to know about making your underlying data available (as you will be aware, this is required for publication), please see the end of this email. If your institution or institutions have a press office, please notify them about your upcoming article at this point, to enable them to help maximise its impact. Inform journal staff as soon as possible if you are preparing a press release for your article and need a publication date.

Thank you again for supporting open-access publishing; we are looking forward to publishing your work in PLOS Genetics!

Yours sincerely,

Stephen Bush

Academic Editor

PLOS Genetics

Giorgio Sirugo

Section Editor

PLOS Genetics

Aimée Dudley

Editor-in-Chief

PLOS Genetics

Anne Goriely

Editor-in-Chief

PLOS Genetics

www.plosgenetics.org

BlueSky: @plos.bsky.social

----------------------------------------------------

Comments from the reviewers (if applicable):

This paper was originally reviewed by four reviewers, of whom one made a recommendation to accept outright, and three others suggesting revisions. The authors have made a comprehensive reply to reviewer comments – inclusive of additional immunofluorescence experiments, tempering claims throughout the text and the addition of a dedicated ‘limitations’ section – with their revised manuscript sent back to all four original reviewers. However, only two of these reviewers agreed to re-review the manuscript. The reviewer who had originally recommend acceptance declined to see it again; one other reviewer (original decision: ‘major revision’) did not respond. At this stage, three of the four original reviewers have recommended acceptance. I am of the view that the authors have addressed the comments made by the sole original reviewer who has not seen the revision, too, and on this basis recommend that this manuscript is accepted for publication.

Reviewer's Responses to Questions

**Comments to the Authors:**

**Please note here if the review is uploaded as an attachment.**

Reviewer #1: I appreciate the authors’ careful attention to my previous feedback. The revisions have noticeably enriched the manuscript. By detailing the background of the DT and DSE breeds and clarifying the central hypothesis, the introduction now sets a much stronger stage for the research. Additionally, the expanded discussion on isoform complexity and the physiological implications of the enriched pathways adds valuable depth to the study. I am also glad to see the dataset is now properly deposited.

The authors have fully resolved all of my concerns, and the manuscript now reads exceptionally well. I am pleased to recommend this valuable work for publication.

Reviewer #3: I have carefully reviewed the revised manuscript and the authors' point-by-point responses. The authors have thoroughly and satisfactorily addressed all the concerns raised in my initial review.

The key issues—particularly the distinction between altitude adaptation and genetic background, the clarification of computational methods, the framing of inferred results, and the typographical errors—have all been properly resolved. The manuscript's scientific rigor and readability are significantly improved, and the conclusions are now well-supported by the data.

I have no further comments or concerns. I recommend the manuscript for acceptance and publication in its current form.

**********

**Have all data underlying the figures and results presented in the manuscript been provided?**

Large-scale datasets should be made available via a public repository as described in the *PLOS Genetics*  data availability policy, and numerical data that underlies graphs or summary statistics should be provided in spreadsheet form as supporting information.

Reviewer #1: Yes

Reviewer #3: Yes

**********

PLOS authors have the option to publish the peer review history of their article (what does this mean?). If published, this will include your full peer review and any attached files.

If you choose “no”, your identity will remain anonymous but your review may still be made public.

**Do you want your identity to be public for this peer review?**  For information about this choice, including consent withdrawal, please see our Privacy Policy.

Reviewer #1: No

Reviewer #3: No

----------------------------------------------------

**Data Deposition**

If you have submitted a Research Article or Front Matter that has associated data that are not suitable for deposition in a subject-specific public repository (such as GenBank or ArrayExpress), one way to make that data available is to deposit it in the Dryad Digital Repository. As you may recall, we ask all authors to agree to make data available; this is one way to achieve that. A full list of recommended repositories can be found on our website.

The following link will take you to the Dryad record for your article, so you won't have to re‐enter its bibliographic information, and can upload your files directly:

http://datadryad.org/submit?journalID=pgenetics&manu=PGENETICS-D-26-00113R1

More information about depositing data in Dryad is available at http://www.datadryad.org/depositing. If you experience any difficulties in submitting your data, please contact help@datadryad.org for support.

Additionally, please be aware that our data availability policy requires that all numerical data underlying display items are included with the submission, and you will need to provide this before we can formally accept your manuscript, if not already present.

----------------------------------------------------

**Press Queries**

If you or your institution will be preparing press materials for this manuscript, or if you need to know your paper's publication date for media purposes, please inform the journal staff as soon as possible so that your submission can be scheduled accordingly. Your manuscript will remain under a strict press embargo until the publication date and time. This means an early version of your manuscript will not be published ahead of your final version. PLOS Genetics may also choose to issue a press release for your article. If there's anything the journal should know or you'd like more information, please get in touch via plosgenetics@plos.org.

# Editor report: Acceptance letter

**DOI:** 10.1371/journal.pgen.1012267.r004

**Authors:** Giorgio Sirugo, Stephen Bush

PGENETICS-D-26-00113R1

Integrating short-read and long-read single-cell transcriptomics of pig pituitary reveals mechanisms of high-altitude hypoxia adaptation

Dear Dr Huo,

We are pleased to inform you that your manuscript entitled "Integrating short-read and long-read single-cell transcriptomics of pig pituitary reveals mechanisms of high-altitude hypoxia adaptation" has been formally accepted for publication in PLOS Genetics! Your manuscript is now with our production department and you will be notified of the publication date in due course.

The corresponding author will soon be receiving a typeset proof for review, to ensure errors have not been introduced during production. Please review the PDF proof of your manuscript carefully, as this is the last chance to correct any errors. Please note that major changes, or those which affect the scientific understanding of the work, will likely cause delays to the publication date of your manuscript.

Soon after your final files are uploaded, unless you have opted out or your manuscript is a front-matter piece, the early version of your manuscript will be published online. The date of the early version will be your article's publication date. The final article will be published to the same URL, and all versions of the paper will be accessible to readers.

For Research Articles, you will receive an invoice from PLOS for your publication fee after your manuscript has reached the completed accept phase. If you receive an email requesting payment before acceptance or for any other service, this may be a phishing scheme. Learn how to identify phishing emails and protect your accounts at https://explore.plos.org/phishing.

Thank you again for supporting PLOS Genetics and open-access publishing. We are looking forward to publishing your work!

With kind regards,

Sharmila Kamatchi

PLOS Genetics

On behalf of:

The PLOS Genetics Team

Carlyle House, Carlyle Road, Cambridge CB4 3DN | United Kingdom

plosgenetics@plos.org | +44 (0) 1223-442823

plosgenetics.org | Twitter: @PLOSGenetics
